# Elucidating the Genetic Basis of Columnar Upright Architecture in *Populus* Through CRISPR Disruption of *TILLER ANGLE CONTROL1*

**DOI:** 10.1111/pbi.70415

**Published:** 2025-10-22

**Authors:** Na‐Young Choi, Min‐Ha Kim, Hyun‐A Jang, Seung‐Won Pyo, Kong‐Young Park, Hyoshin Lee, Eun‐Kyung Bae, Jae‐Heung Ko

**Affiliations:** ^1^ Department of Convergent Biotechnology and Advanced Materials Science and Graduate School of Green‐Bio Sciences Kyung Hee University Yongin Republic of Korea; ^2^ Department of Forest Bio‐resources National Institute of Forest Science Suwon Republic of Korea; ^3^ Uriseed Company Icheon Republic of Korea

**Keywords:** auxin, columnar architecture, CRISPR gene editing, far‐red light, gravitropic response, hybrid poplar, Lombardy poplar, tiller angle control1

## Abstract

Narrow or upright branch angles in shoots and leaves lead to columnar, upright‐growing tree architectures, as observed in various tree species such as Lombardy poplar ( *Populus nigra* var. *italica* ). However, the genetic mechanism underlying this unique growth habit in Lombardy poplar has not yet been elucidated. In this study, we identified a nonsense mutation in the *PnTAC1‐1* gene of Lombardy poplar, an ortholog of the rice *TILLER ANGLE CONTROL 1* (*TAC1*) gene known to regulate branch angles. To confirm the functional role of *TAC1* in regulating tree architecture, we generated transgenic hybrid poplar ( *Populus alba* × *Populus glandulosa*, clone BH) with targeted mutations in *TAC1* homologues using CRISPR/Cas9 gene editing. The resulting *TAC1‐CRISPR* hybrid poplars exhibited a stable upright branching phenotype closely resembling that of Lombardy poplar, as confirmed by two consecutive years of living modified organism (LMO) field trials. Anatomical analysis revealed increased cell elongation specifically in the lower petiole region and significantly enhanced gravitropic responses in *TAC1‐CRISPR* hybrid poplars compared to wild‐type BH clones. RNA sequencing analysis further demonstrated that *TAC1* disruption triggered extensive transcriptomic reprogramming of axillary meristem, notably altering hormonal and photomorphogenic signalling pathways, which redirected auxin accumulation toward the abaxial region and increased gibberellin biosynthesis, ultimately promoting upright growth. This research uncovers the genetic and molecular mechanisms behind columnar growth in poplar and provides a promising approach for engineering tree architecture to enhance planting density, harvest efficiency and woody biomass productivity.

## 1 Introduction

Lombardy poplars ( *Populus nigra* var. *italica* ), selected in the Lombardy region of northern Italy in the 19th century, are tall, slender and fast‐growing trees. They are best known for their columnar form and unusual branching structure; the branches grow upward, parallel to the trunk (Zsuffa 1974). This unique growth form has popularised Lombardy poplars as a natural ‘living wall’ for rapid privacy screening. Lombardy poplars are distributed from Europe to worldwide through vegetative propagation as a single male genotype (Zsuffa 1974) and were introduced to Korea in the early 1900s (Cagelli and Lefevre 1995).

Plant architecture, encompassing shoot branching, organ shape and overall spatial organisation, plays a fundamental role in ecological adaptation and resource efficiency. Slight modifications in architectural traits, particularly in lateral shoot orientation, can influence how plants intercept light, compete for space and optimise photosynthetic efficiency (Reinhardt and Kuhlemeier 2002; Hollender et al. 2020; Basu and Parida 2023). One such adaptive response is the Shade Avoidance Syndrome (SAS), wherein plants exposed to low red‐to‐far‐red light ratios undergo elongation of shoots and petioles, driven by auxin accumulation and redistribution (Yang and Li 2017; Ma and Li 2019). These changes enable vertical growth that enhances access to light. However, broad or spreading branch angles require more planting space, which is not ideal for forestry and biomass production. In contrast, columnar trees with upright architecture allow higher planting densities, improve light penetration into the canopy and reduce harvesting costs (Burgess et al. 2017; Mantilla‐Perez and Salas Fernandez 2017; Fladung 2021). This growth form has been observed in several tree species, including the ‘pillar’ peach, where a mutation in *PpeTAC1*, the ortholog of rice *TILLER ANGLE CONTROL 1* (*TAC1*), results in reduced branch angles and vertical shoot growth (Dardick et al. 2013; Hollender, Waite, et al. 2018).

*TAC1* expression is generally upregulated under optimal light conditions and suppressed under shade, leading to more vertical branch orientation (Hollender, Waite, et al. 2018; Basu and Parida 2023). Functional studies in plum have shown that RNAi‐mediated silencing of *TAC1* recapitulates the upright architecture seen in ‘pillar’ peach, while its overexpression produces wider, horizontal branching (Hollender, Waite, et al. 2018). Based on this, it has been hypothesized that the columnar growth of Lombardy poplar may be due to a mutation in its *TAC1* ortholog, though this had not been experimentally validated. Recently, Fladung (2021) confirmed the role of *TAC1* by generating pyramidal hybrid poplars (*Populus* × *canescens*) through CRISPR/Cas9‐mediated knockout of *TAC1* genes. Notably, the disruption of just one (*TAC‐14*, Potri.014G102600) of the two *TAC1* homologs was sufficient to induce a columnar phenotype. More recently, similar gene‐editing approaches in citrus and cotton have further demonstrated that *TAC1* knockout reduces both petiole and branch angles, reinforcing the conserved role of TAC1 in shoot orientation across species (Dutt et al. 2022; Kangben et al. 2024).

In addition to *TAC1*, the IGT gene family includes *LAZY* and *DEEPER ROOTING* (*DRO*) subfamilies, which regulate shoot angle via gravitropic responses and auxin redistribution (Duvick 2005; Yu et al. 2007; Ku et al. 2011; Li et al. 2017). While TAC1 promotes wider branch angles in response to light cues, LAZY1 restricts lateral growth by enhancing auxin flow toward the lower side of branches, thus promoting upright growth (Waite and Dardick 2021). Although LAZY and DRO pathways are well characterised, the specific molecular mechanisms by which TAC1 exerts its function remain less clear. Some evidence suggests that TAC1 may negatively regulate LAZY1, though their relationship appears to be more complex and indirect (Hollender et al. 2020; Dutt et al. 2022).

In this study, we identified a nonsense mutation in the *PnTAC1‐1* gene of Lombardy poplar. To elucidate the functional role of *TAC1* in tree architecture, we employed CRISPR/Cas9‐mediated gene editing to disrupt all *TAC1* homologues in hybrid poplar ( *Populus alba* × *Populus glandulosa*, clone BH). The resulting *TAC1‐CRISPR* hybrid poplars displayed a stable upright branching phenotype, closely resembling that of Lombardy poplar, as confirmed through multi‐season LMO field trials. Interestingly, these gene‐edited trees exhibited enhanced gravitropic responses without changes in *PtrLAZY1* expression. Transcriptome analyses revealed major shifts in gene expression related to far‐red light signalling and auxin biosynthesis, implicating altered auxin accumulation and distribution as a key driver of the upright architecture. Taken together with prior work, our results strongly implicate *TAC1* in the columnar phenotype of Lombardy poplar and confirm *TAC1* as a promising target for engineering poplar architecture to improve silvicultural efficiency and biomass yield.

## 2 Results

### 2.1 Lombardy Poplar Has a Nonsense Mutation in *PnTAC1‐1* Gene

**FIGURE 1:**
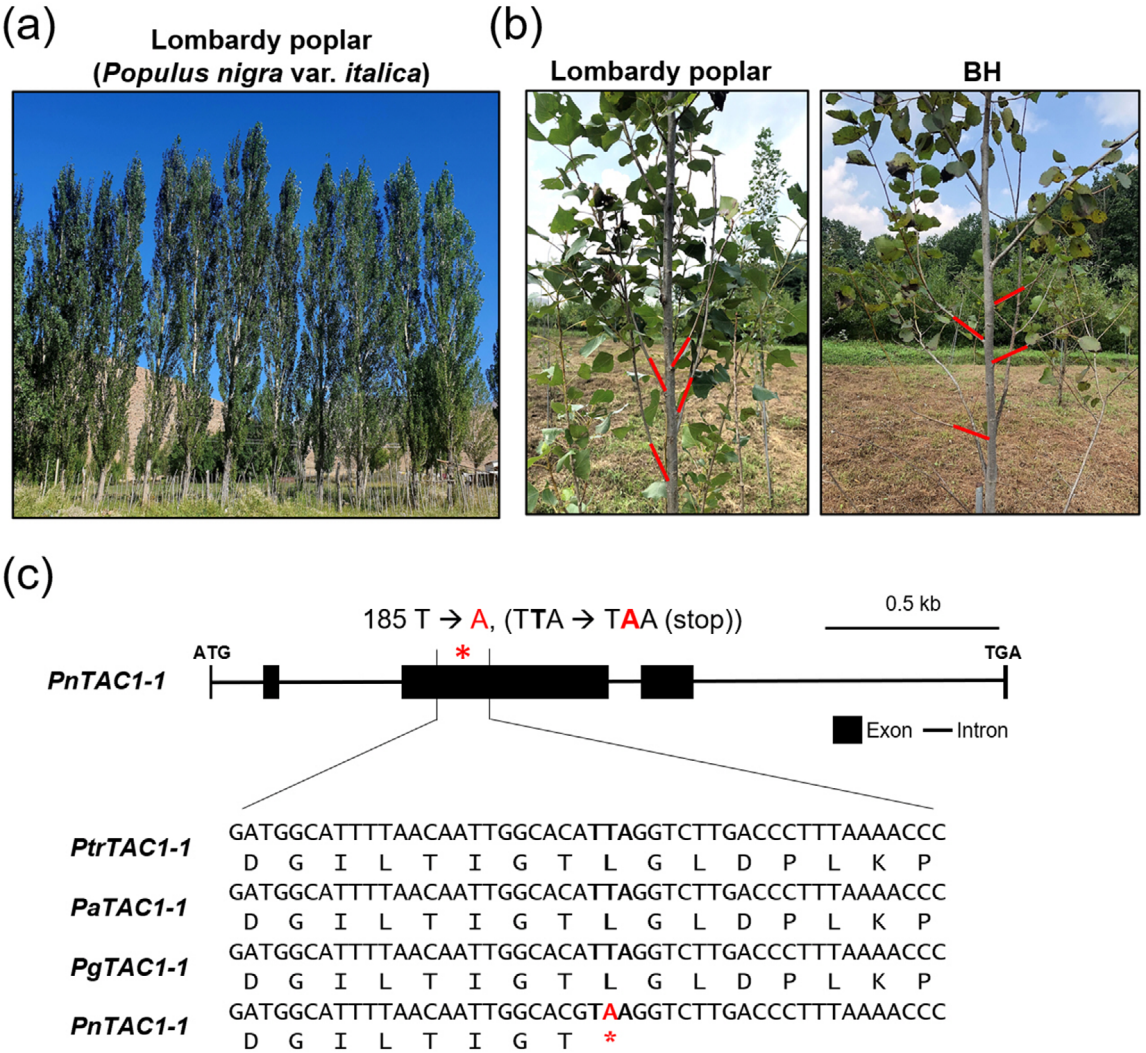
Lombardy poplar has a non‐sense mutation in the *PnTAC1‐1* gene. (a) Lombardy poplar ( *P. nigra* var. *italica* ) showing its characteristic upright, columnar growth habit in the field. (b) Comparison between Lombardy poplar (left) and the hybrid poplar clone BH ( *P. alba* × *P. glandulosa* , clone BH) grown at the Forest Bioresources Department of NIFoS, Republic of Korea. Red arrows indicate representative branch orientations, showing markedly steeper, upright branches in Lombardy poplar relative to BH. (c) Gene structure and sequence alignment of *TAC1* homologues. A schematic of the *PnTAC1‐1* gene shows a point mutation (T → A) in the third exon, converting a leucine codon (TTA) into a premature stop codon (TAA), likely resulting in a nonfunctional protein. Aligned nucleotide and amino acid sequences of *TAC1‐1* from *P. trichocarpa* (*PtrTAC1‐1*), *P. alba* (*PaTAC1‐1*), *P. glandulosa* (*PgTAC1‐1*) and *P. nigra* var. *italica* (*PnTAC1‐1*) highlight the nonsense mutation in red with an asterisk.

Lombardy poplar ( *P. nigra* var. *italica* ) exhibits a distinctive columnar growth habit, characterised by steep, upright branching (Figure 1a,b). Although this phenotype has long been hypothesized to result from mutations in a *TAC1* ortholog (Fladung 2021), direct genetic evidence has been lacking.

To investigate this hypothesis, we sequenced the genomic regions corresponding to *PnTAC1‐1* and *PnTAC1‐2* in two independent trees of male Lombardy poplar clone grown at the Forest Bioresources Department of the National Institute of Forest Science (NIFoS), Republic of Korea (latitude 37.2° N, longitude 126.9° E) (Figure 1b). It has been reported that *Populus* species have two *TAC1* homologues; *P. trichocarpa* has *PtrTAC1‐1* (Potri.014G102600) and *PtrTAC1‐2* (Potri.002G175300), which have greater than 90% amino acid sequence similarity (Fladung 2021; Figure S1). Indeed, we have found a nonsense mutation in the third exon of the *PnTAC1‐1* gene in both sampled Lombardy poplar trees (Figure 1c; Figure S2). The point mutation (185T to A) results in a nonsense mutation (TAA, stop codon) and is homozygous in both sampled Lombardy poplar trees, likely causing a complete loss‐of‐function of *PnTAC1‐1* (Figure 1c; Figure S1). However, no mutations were found in *PnTAC1‐2* of both clones (data not shown). These results indicate that the nonsense mutation in *PnTAC1‐1* may underlie the columnar growth habit of Lombardy poplar, consistent with the finding that *TAC1‐4* in *Populus* × *canescens*—orthologous to *PnTAC1‐1* in *P. nigra* and *PtrTAC1‐1* (Potri.014G102600) in *P. trichocarpa* —is sufficient to confer a similar phenotype (Fladung 2021).

### 2.2 Production of Transgenic Hybrid Poplars With CRISPR‐Knockout of *TAC1* Genes

**FIGURE 2:**
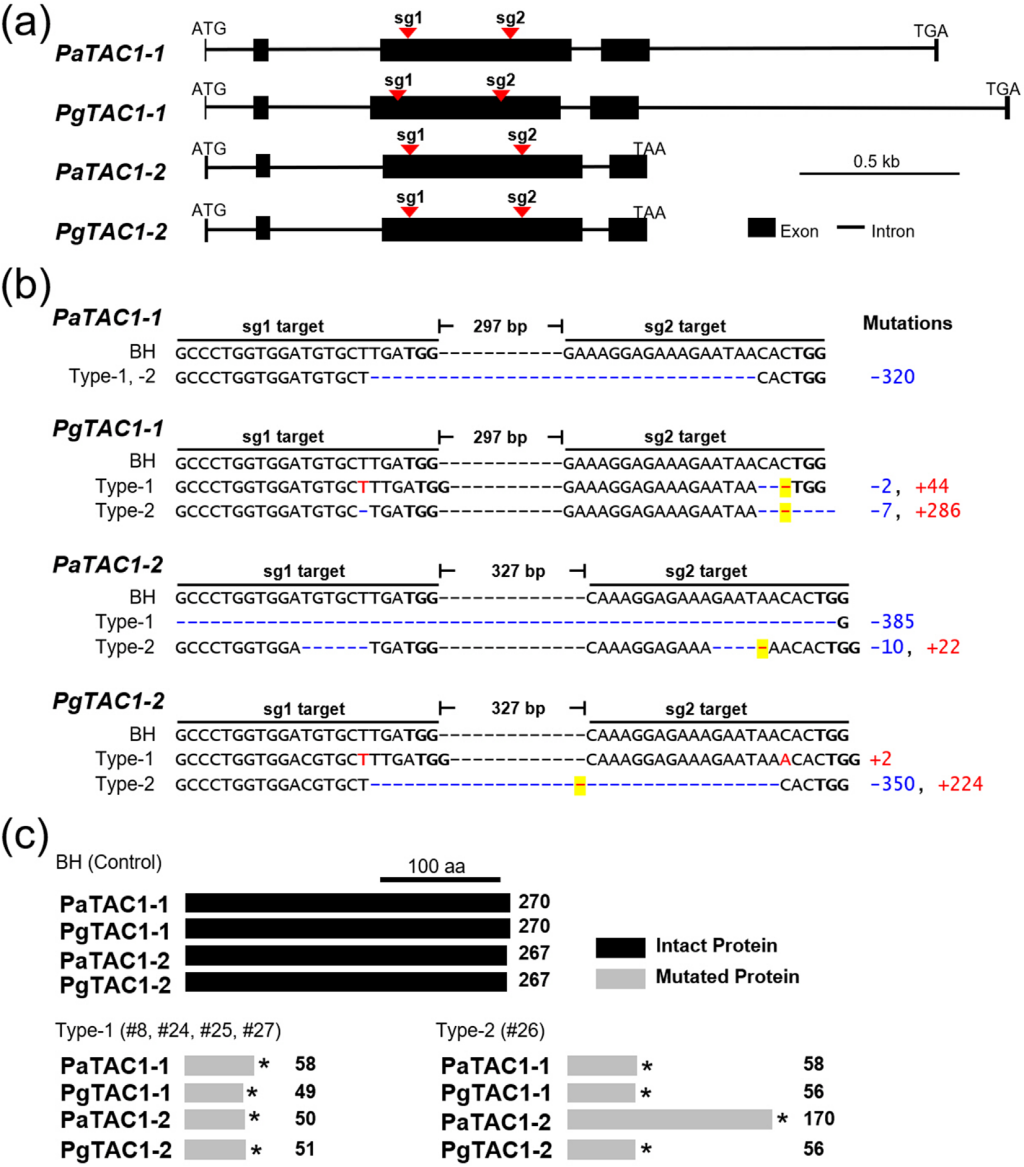
Generation of CRISPR/Cas9‐mediated knockout lines targeting *TAC1* homologues in hybrid poplar. (a) Gene structures of *TAC1* homologues from *P. alba* (*PaTAC1‐1* and *PaTAC1‐2*) and *P. glandulosa* (*PgTAC1‐1* and *PgTAC1‐2*) showing the locations of two single guide RNA (sg1 and sg2) target sites used for genome editing (Figure S3). Black boxes represent exons and lines indicate introns. (b) CRISPR/Cas9‐induced indel mutations in selected transgenic lines. Type‐1 lines (#8, #24, #25, #27) and type‐2 line (#26) exhibit biallelic mutations at the sg1 and sg2 target sites. PAM sequences are in bold; deletions are shown as blue dashes, insertions as red text and large insertions are indicated by yellow‐highlighted sequences. Mutation sizes (deletions in blue, insertions in red) are listed at the right. (c) Summary of predicted TAC1 protein lengths based on mutation profiles. Black bars represent full‐length TAC1 proteins in the wild‐type BH clone, while grey bars indicate truncated proteins in mutant lines. Asterisks denote premature stop codons resulting from nonsense mutations, with amino acid lengths shown to the right.

The hybrid poplar ( *P. alba* × *P. glandulosa* , clone BH) used in this study carries two distinct sets of *TAC1* homologues: *PaTAC1‐1* and *PaTAC1‐2* from the *P. alba* genome and *PgTAC1‐1* and *PgTAC1‐2* from the *P. glandulosa* genome (Figure S1). To disrupt all four *TAC1* alleles simultaneously using CRISPR/Cas9‐mediated genome editing, two single‐guide RNAs (sg1 and sg2) were designed to target the third exon of each gene, with sgRNA target sites spaced 297 bp apart in *TAC1‐1* homologues and 327 bp apart in *TAC1‐2* homologues (Figure 2a; Figure S3). Among the 27 regenerated lines, five (#8, #24, #25, #26 and #27) were confirmed as transgenic (Figure S4) and subjected to detailed analysis using the Illumina MiniSeq platform, which revealed biallelic insertions and deletions (indels) in all four *TAC1* genes (Figure 2b). Gene structure analysis of the edited alleles (Figure S5) revealed that all mutations led to frameshifts or premature stop codons in the third exon, resulting in truncated TAC1 proteins with a minimum predicted length of 49 amino acids (Figure 2c). These alterations are expected to produce loss‐of‐function alleles in all edited lines, supporting their use in downstream functional analyses.

Based on the mutation patterns, the lines were classified into two categories: Type‐1, comprising lines #8, #24, #25 and #27, and Type‐2, represented by line #26 (Figure 2b). All five lines exhibited large indel mutations within the targeted exon, consistent with previous findings that closely spaced sgRNA targets can result in substantial deletions (Do et al. 2019). Interestingly, the four Type‐1 lines (#8, #24, #25 and #27) displayed identical mutation patterns. Although these shoots were regenerated from spatially separated stem tissues and maintained independently, it remains possible that they originated from a single transformation event followed by clonal propagation. At the same time, identical indel patterns across independent CRISPR/Cas9 lines are not unexpected, as double‐strand breaks are frequently repaired through preferred non‐homologous end joining (NHEJ) pathways, which can recurrently produce the same mutation at a given target site (Chakrabarti et al. 2019; Brant et al. 2024). A definitive test of independence will require analysis of T‐DNA insertion sites, for example using TAIL‐PCR or whole‐genome sequencing. Pending such confirmation, we conservatively designated lines #8, #24, #25 and #27 as a single Type‐1 event and line #26 as a distinct Type‐2 event.

To ensure robust interpretation, all major phenotypic and molecular analyses in this study were therefore conducted using one representative Type‐1 line (#24) together with the independent Type‐2 line (#26).

### 2.3 Narrow Petiole and Branch Angles in Transgenic *TAC1‐CRISPR* Hybrid Poplars Lead to an Upright Growth Architecture

**FIGURE 3:**
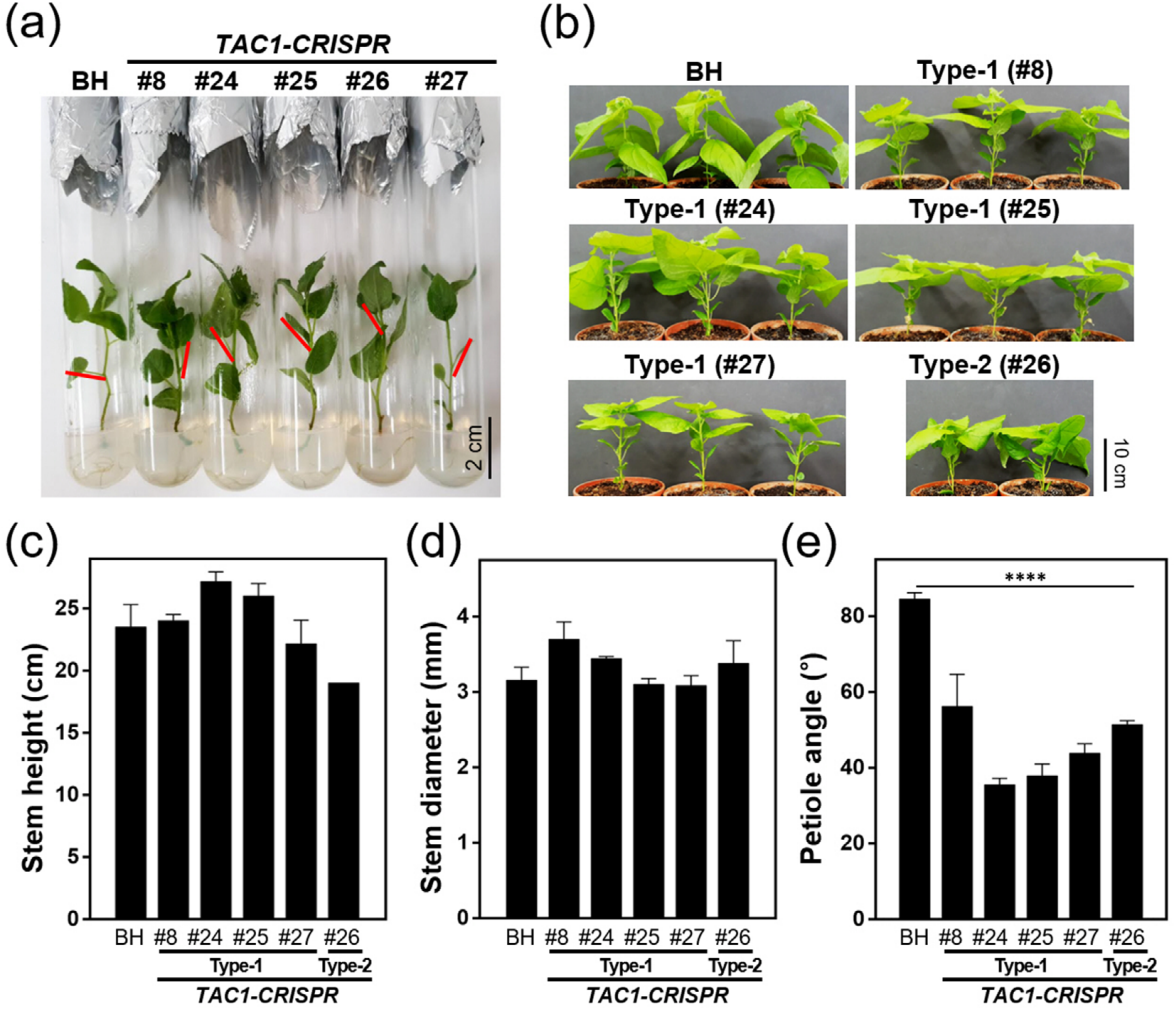
Transgenic *TAC1‐CRISPR* hybrid poplars exhibit an upright growth habit with narrow petiole angles. (a) Five‐week‐old *TAC1‐CRISPR* hybrid poplars grown in test tubes after rooting. Red arrows indicate petiole angles relative to the stem, highlighting the narrower angles in all mutant lines compared to BH. (b) Eight‐week‐old soil‐acclimated *TAC1‐CRISPR* lines show a consistent upright leaf orientation with reduced petiole angles. (c–e) Quantitative analysis of 12‐week‐old soil‐acclimated plants. While stem height (c) and stem diameter (d) remained comparable between *TAC1‐CRISPR* lines and BH controls, petiole angle (e) was significantly reduced in all transgenic lines, confirming the altered shoot architecture. Data represent mean ± SD (*n* = 5). Statistical significance was determined using one‐way ANOVA followed by post hoc comparisons between BH and *TAC1‐CRISPR* lines, with asterisks indicating significant differences (****p < 0.0001).

**FIGURE 4:**
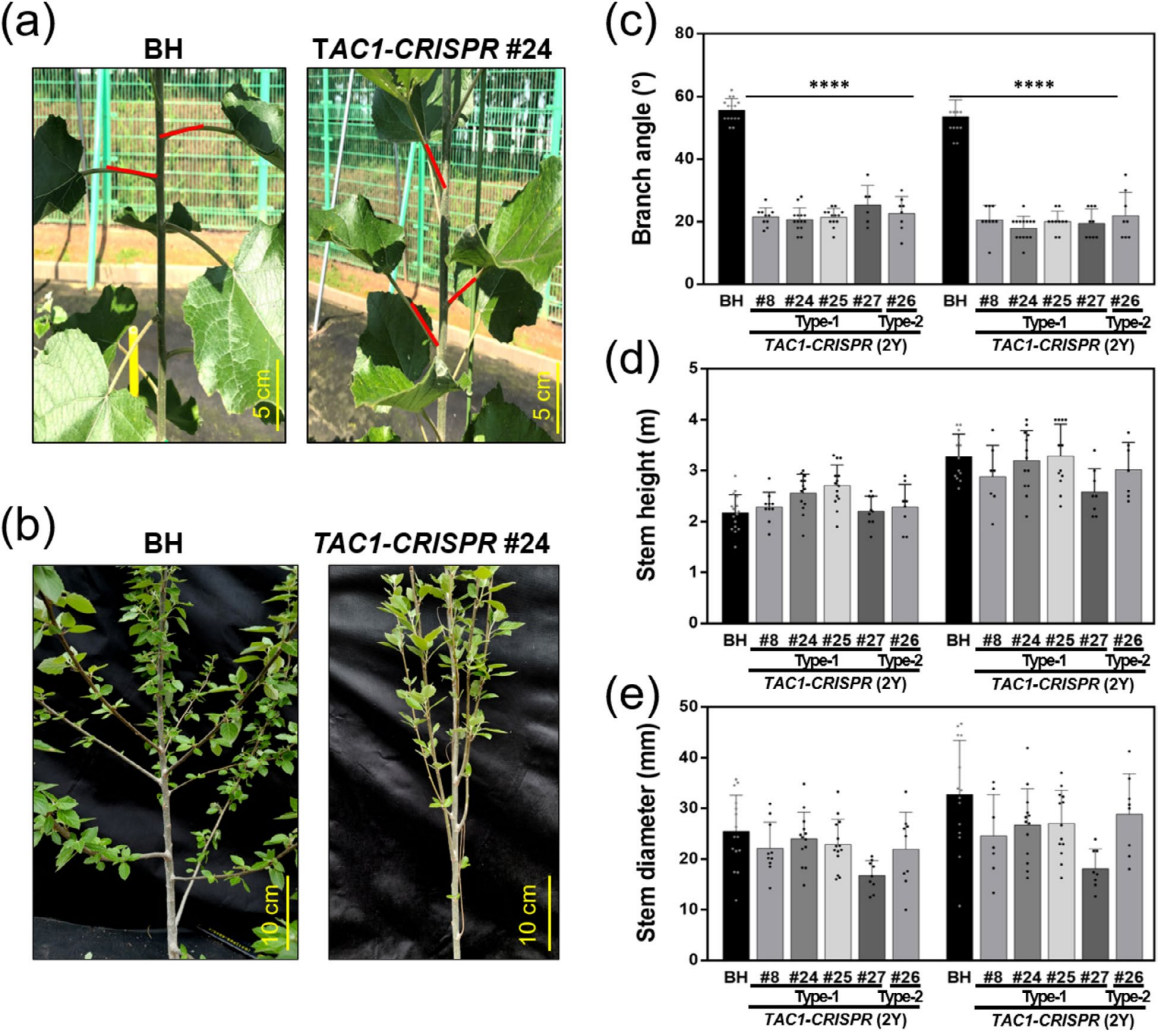
Narrow‐angled, upright growth architecture of *TAC1‐CRISPR* hybrid poplars in LMO field trials. (a) Five‐month‐old *TAC1‐CRISPR* hybrid poplar line #24 grown in the LMO field shows markedly reduced petiole angles compared to wild‐type BH. Red lines highlight petiole orientation. Photograph taken on September 10, 2021. (b) After 12 months of growth, *TAC1‐CRISPR* hybrid poplars maintain narrow branch angles, consistent with an upright growth phenotype. Photograph taken on April 25, 2022. (c–e) Quantitative analysis of branch angle (c), stem height (d) and stem diameter (e) at the end of each growing season (1Y: December 6, 2021; 2Y: October 28, 2022). *TAC1‐CRISPR* lines exhibited significantly narrower branch angles compared to BH, while stem height and diameter remained unaffected. Data were analysed using one‐way ANOVA followed by Tukey's multiple comparison test (GraphPad Prism). Values are presented as mean ± SD, with statistical significance indicated as ****p < 0.0001.

*TAC1‐CRISPR* hybrid poplars exhibited a distinct upright growth habit, characterised by significantly narrower petiole and branch angles compared to wild‐type BH plants. Phenotypic differences were apparent from the earliest growth stages in test‐tube cultures, where all *TAC1‐CRISPR* lines, including Type‐1 event (#8, #24, #25 and #27) and the Type‐2 event (#26), exhibited reduced petiole angles relative to BH (Figure 3a). After 8 weeks of soil acclimation, this phenotype remained consistent, with mutant lines maintaining steeper petiole orientations (Figure 3b). Quantitative measurements taken at 12 weeks confirmed a significant reduction in petiole angle in *TAC1‐CRISPR* lines (BH: 48.2° ± 3.1°, *n* = 5; #24: 26.4° ± 2.8°, *p* < 0.001; #26: 27.1° ± 3.0°, *p* < 0.001), while no notable differences were observed in stem height or diameter (Figure 3c–e). To assess whether these traits were maintained under field conditions, a long‐term evaluation was conducted in a Living Modified Organism (LMO) field trial. Plants were transplanted on May 2, 2021, and monitored through seasonal growth cycles. By 5 months post‐transplantation, the narrow petiole angle phenotype persisted in line #24 (Figure 4a), and after 12 months, field‐grown *TAC1‐CRISPR* trees showed clearly narrower branch angles than BH controls (Figure 4b). Quantitative analysis at the end of each growing season (year 2021 and 2022) confirmed that the upright architecture was stable and reproducible across field‐grown plants (Figure 4c). Importantly, stem height and diameter in *TAC1‐CRISPR* hybrid poplars remained comparable to BH throughout the evaluation period, including post‐winter assessments (Figure 4d,e).

In a parallel experiment, *TAC1‐CRISPR* hybrid poplars were generated using a CRISPR/Cas9 geminiviral replicon vector (pPLID4), which was used to produce transgene‐free edited tomato plants (Vu et al. 2020). Among 40 regenerated plants, genomic DNA‐PCR identified *TAC1* edits in 17/40 (42.5%) (Figure S6a). Four representative lines (#17, #22, #31, #36) carried frameshifting mutations predicted to abolish *TAC1* function (Figure S6b,c). These edited plants, when transplanted to the LMO site, exhibited reduced branch angles relative to BH controls, while stem height and diameter remained unaffected (Figure S7).

Together, these results demonstrate that CRISPR‐mediated disruption of *TAC1* genes in hybrid poplar effectively alters shoot architecture by reducing petiole and branch angles, resulting in an upright growth form without negatively impacting overall vegetative growth.

### 2.4 Asymmetric Cell Elongation in Petiole Tissues of *TAC1‐CRISPR* Hybrid Poplars

**FIGURE 5:**
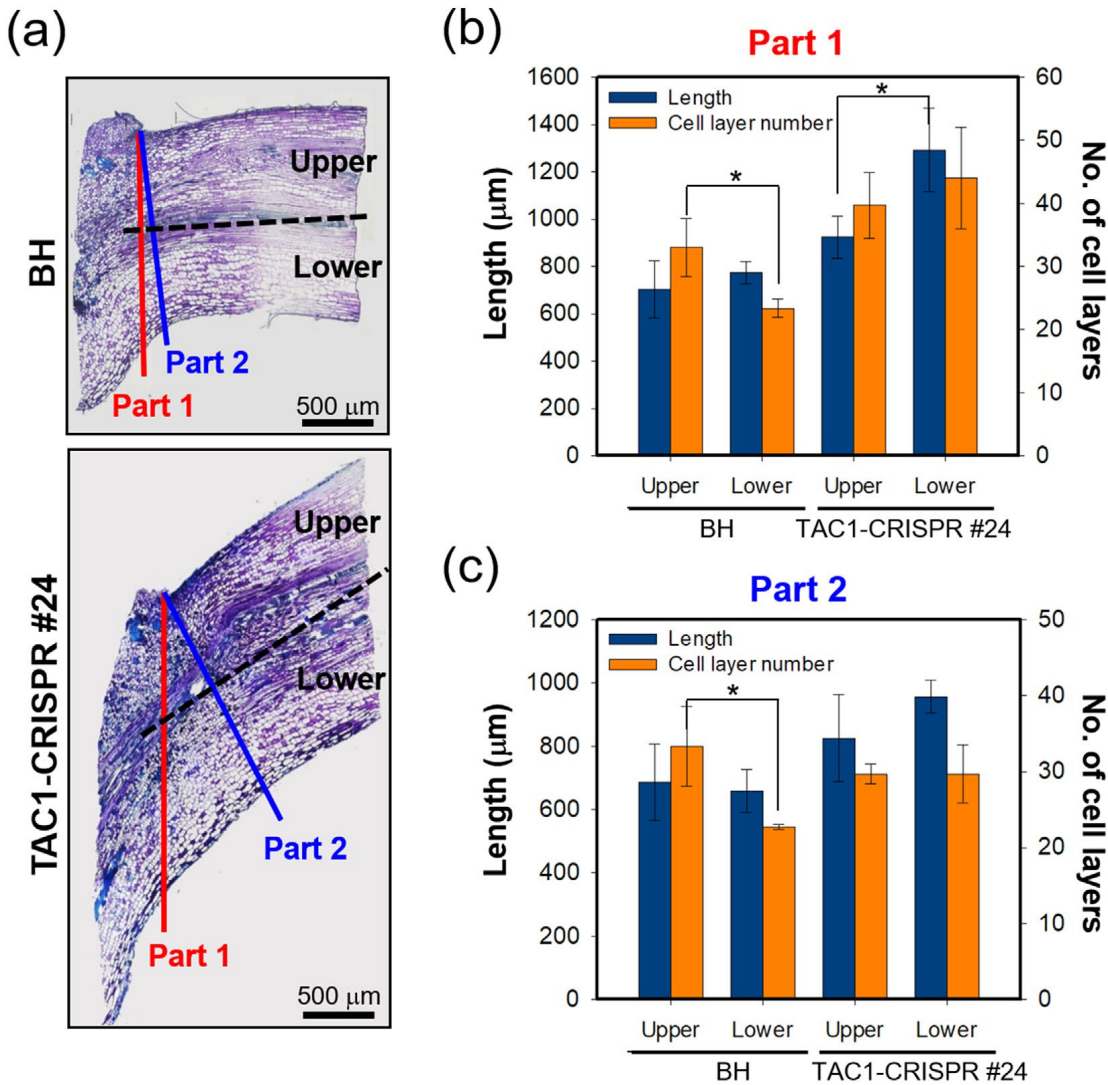
Asymmetric cell elongation in petiole tissues contributes to the upright growth phenotype of *TAC1‐CRISPR* hybrid poplars. (a) Representative longitudinal sections of petioles from BH and *TAC1‐CRISPR* #24 hybrid poplar. Red (Part 1) and blue (Part 2) lines indicate two measurement orientations: Part 1 aligns with the petiole‐stem attachment axis, and Part 2 is perpendicular to it. Dashed lines divide the upper and lower regions of the petiole used for analysis. Scale bars = 500 μm. (b, c) Quantitative comparisons of cell length (blue bars) and cell layer number (orange bars) in upper and lower regions of Part 1 (b) and Part 2 (c). In Part 1, *TAC1‐CRISPR* hybrid poplars exhibit significantly increased length in the lower region with no change in cell number, suggesting enhanced cell elongation rather than increased cell division. Statistical analysis was performed using Student's *t*‐test to compare upper and lower regions within each genotype. Data are presented as mean ± SD, and asterisks indicate significant differences at *p* < 0.05 (*n* = 3).

To investigate the cellular basis of the upright leaf orientation in *TAC1‐CRISPR* hybrid poplars, we performed a comparative anatomical analysis of petiole longitudinal sections from BH and *TAC1‐CRISPR* #24 (type‐1) (Figure 5). Line #24 was chosen as a representative knockout because all five edited poplars carried biallelic loss‐of‐function mutations in all *TAC1* homologues and displayed an indistinguishable reduced branch angle phenotype (Figure 4). Thus, the anatomical features observed in *TAC1‐CRISPR* #24 (Type‐1) are considered representative of the entire knockout set. Petioles were divided into upper and lower regions using the central vascular strand as a reference, and measurements were taken in two planes: Part 1, aligned with the petiole–stem junction, and Part 2, perpendicular to this axis (Figure 5a). In BH petioles, Part 1 showed a higher number of cell layers in the upper region than in the lower region, with no significant difference in regional length. In contrast, *TAC1‐CRISPR* #24 petioles displayed a reversed pattern in Part 1, with the lower region significantly longer than the upper, while cell layer numbers remained unchanged (Figure 5b). In Part 2, BH maintained the pattern of more upper cell layers, whereas no significant difference in either cell length or cell layer number was observed in *TAC1‐CRISPR* #24 (Figure 5c).

These observations suggest that in *TAC1‐CRISPR* hybrid poplars, localised cell elongation—rather than increased cell proliferation—occurs predominantly in the lower region of the petiole, particularly along the axis connecting the petiole to the stem. This asymmetric elongation likely contributes to the upward reorientation of the petiole, supporting the overall upright leaf architecture in *TAC1‐CRISPR* hybrid poplars.

### 2.5 *TAC1‐CRISPR* Hybrid Poplars Showed Enhanced Gravitropic Responses

**FIGURE 6:**
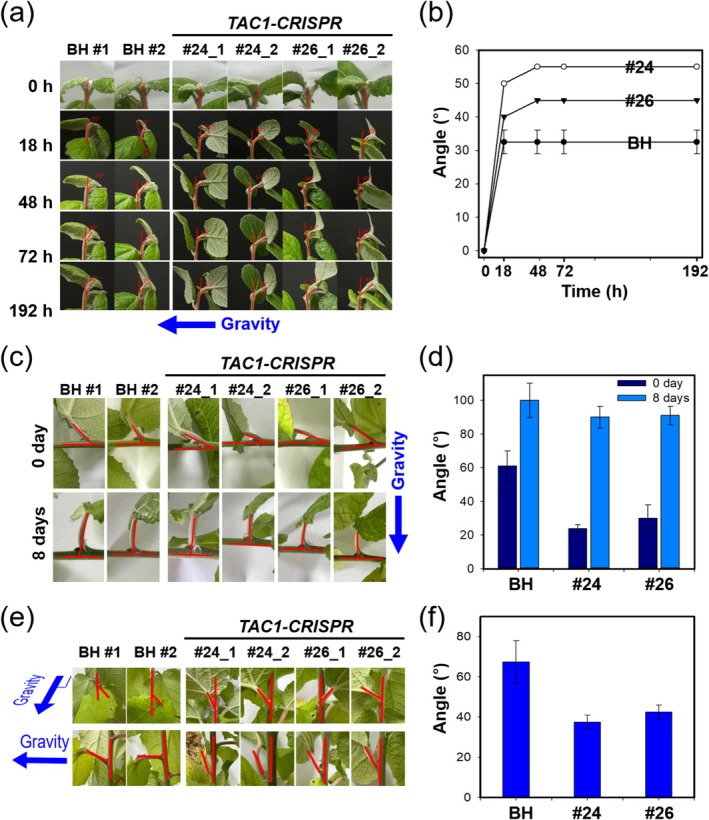
*TAC1‐CRISPR* hybrid poplars display enhanced gravitropic sensitivity and petiole reorientation. (a) Time‐lapse imaging of shoot curvature in BH and *TAC1‐CRISPR* lines (#24 and #26) following 90° horizontal reorientation. Red lines indicate the shoot angles relative to their original vertical orientation. (b) Quantification of shoot angles from (a), showing a faster and steeper gravitropic response in *TAC1‐CRISPR* lines compared to BH. (c–f) Recovery of the eighth petiole angle over 8 days post‐reorientation. Red lines denote petiole angles relative to the stem (c, e). (d) Quantitative comparison of petiole recovery angles at 0 and 8 days from (c). Close‐up images of lower petioles (gravity‐facing side) responding to gravitational stimulus after 8 days (e). (f) Quantitative assessment of petiole angle changes shown in (e), confirming stronger anti‐gravity responses in *TAC1‐CRISPR* lines. Blue arrows indicate the direction of gravity. Error bars represent standard deviation.

To evaluate whether TAC1 influences gravitropic sensitivity, we assessed the response of BH and *TAC1‐CRISPR* lines (#24 and #26) to a 90° reorientation relative to gravity (Figure 6). BH poplars showed only gradual curvature adjustment over time, whereas *TAC1‐CRISPR* hybrid poplars exhibited a more rapid and pronounced gravitropic response, with a significantly sharper shoot angle change observed within the first few hours (Figure 6a,b).

In addition to monitoring shoot reorientation, we examined petiole angle recovery in the 8th leaf over an 8‐day period following gravistimulation. *TAC1‐CRISPR* lines exhibited faster and more complete recovery of petiole angles compared to BH, as confirmed by quantitative measurements of recovery rates (Figure 6c–f). Notably, petioles positioned on the lower, gravity‐facing side of the stem in *TAC1‐CRISPR* plants showed a stronger upward curvature response than those in BH, maintaining significantly steeper angles relative to the stem axis (Figure 6e,f).

Together, the results indicate that disruption of *TAC1* enhances sensitivity to gravity, facilitating quicker and more robust shoot and petiole reorientation. This enhanced gravitropism likely contributes to the development of the upright architecture observed in *TAC1‐CRISPR* hybrid poplars.

### 2.6 *PtrLAZY1* Transcription Remains Unaffected in *TAC1‐CRISPR* Hybrid Poplars

**FIGURE 7:**
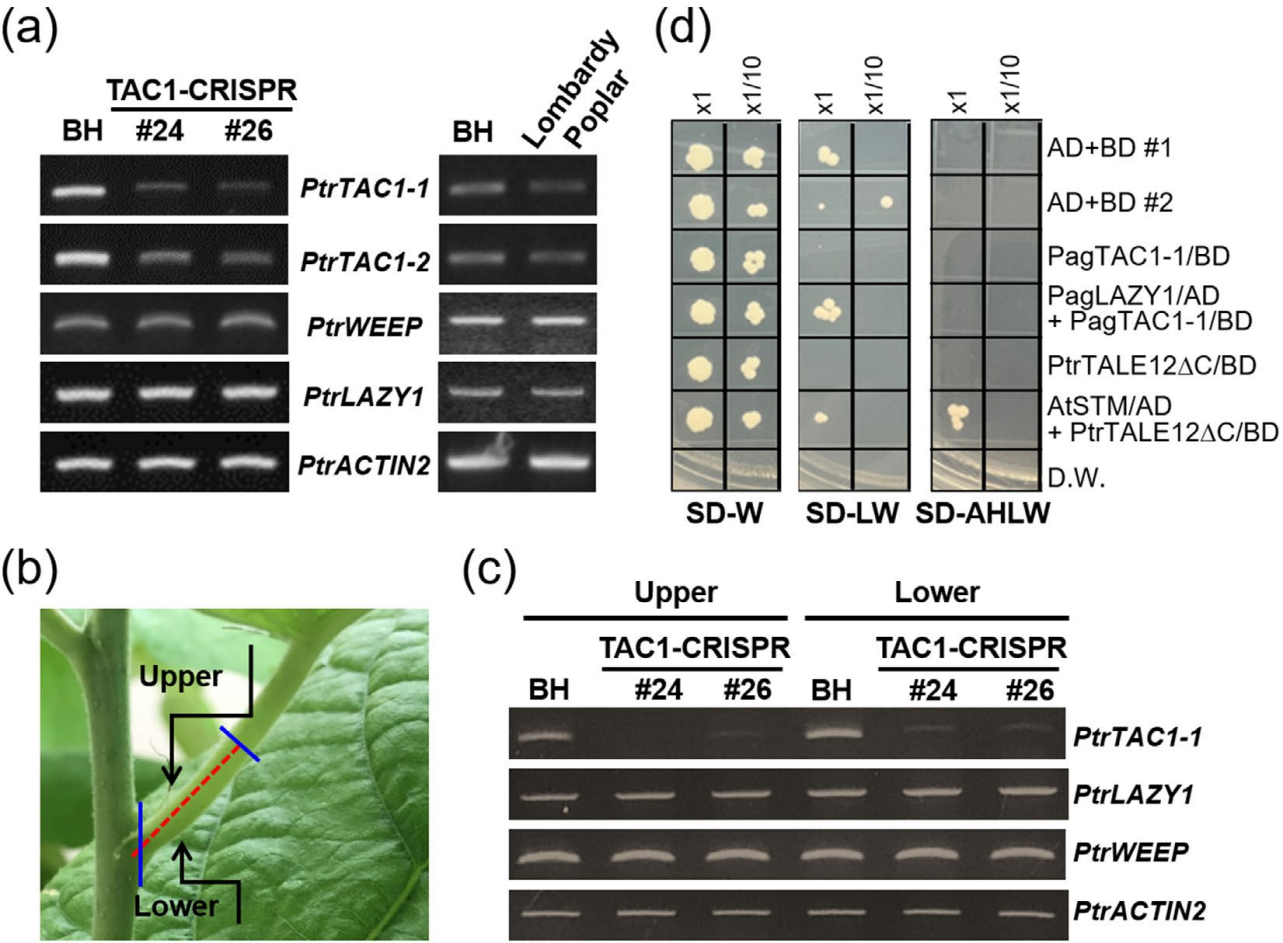
PtrLAZY1 transcription is unaffected in *TAC1‐CRISPR* hybrid poplars, and PtrTAC1 does not interact with PtrLAZY1 at the protein level. (a) Semi‐quantitative RT‐PCR analysis of *PtrTAC1‐1*, *PtrTAC1‐2*, *PtrWEEP* and *PtrLAZY1* expression in BH, *TAC1‐CRISPR* lines (#24 and #26) and Lombardy poplar. *PtrACTIN2* was used as a loading control. (b) Schematic showing the upper and lower petiole regions used for tissue‐specific transcriptional analysis in (c). The upper and lower parts were separated by the red dashed line. (c) Semi‐quantitative RT‐PCR analysis of *PtrTAC1‐1*, *PtrLAZY1* and *PtrWEEP* in the upper and lower petiole tissues of BH and *TAC1‐CRISPR* lines (#24 and #26) with *PtrACTIN2* as a control. (d) Yeast two‐hybrid (Y2H) assay testing protein–protein interaction between PtrTAC1‐1 and PtrLAZY1. Y2H assay was performed by using various combinations of vector constructions (right, see Section 4). AtSTM/AD+PtrTALE12ΔC/BD serves as a positive control (Bae et al. 2020). Yeast was grown on selective media: SD‐W (−Trp), SD‐LW (−Leu, −Trp) and SD‐AHLW (−Ade, –His, −Leu, −Trp). AD and BD represent pGADT7 (activation domain, Leu selection) and pGBKT7 (binding domain, Trp selection) vectors, respectively.

To understand the functional relationship between TAC1 and LAZY, a member of the IGT/LAZY gene family that modulates shoot gravitropism and branch angle across species (Dardick et al. 2013; Hollender, Waite, et al. 2018; Waite and Dardick 2021), we conducted transcriptional and protein interaction studies. In *TAC1‐CRISPR* lines (#24 and #26), the expression of both *PtrTAC1‐1* and *PtrTAC1‐2* was significantly reduced compared to the BH (Figure 7a). Similarly, in Lombardy poplar, which carries a natural mutation in *PnTAC1*, these genes showed reduced expression (Figure 7a). This result aligns with previous reports on our CRISPR poplar studies (Bae et al. 2021; Jang et al. 2021) and is consistent with the mechanism of nonsense‐mediated mRNA decay, a eukaryotic surveillance pathway that eliminates mRNA transcripts containing premature stop codons (Baker and Parker 2004).

To examine transcriptional dynamics, RT‐PCR was performed separately on the upper and lower petiole regions (Figure 7b,c). *PtrWEEP* was included due to its known role in branch angle regulation, with mutations linked to the weeping habit in various species (Hollender, Pascal, et al. 2018; Trozzi 2024). *PtrTAC1‐1* expression was slightly higher in the lower petiole region of BH, supporting its role in promoting downward branch orientation, whereas its expression was strongly reduced in both regions of *TAC1‐CRISPR* lines (#24 and #26), consistent with targeted disruption (Waite and Dardick 2021; Li et al. 2022) (Figure 7c). In contrast, *PtrLAZY* and *PtrWEEP* expression remained uniform across petiole regions and genotypes, including BH, *TAC1‐CRISPR* and Lombardy poplar (Figure 7a,c), suggesting their regulation is independent of *PtrTAC1*.

To examine the interaction between PtrTAC1 and PtrLAZY at the protein level, we performed a yeast two‐hybrid (Y2H) assay (Figure 7d). No interaction was observed between PtrTAC1‐1 and PtrLAZY1, as indicated by the lack of colony growth on selective media (SD‐AHLW). In contrast, the positive controls (AtSTM/AD and PtrTALE12ΔC/BD, Bae et al. 2020) demonstrated successful interactions, confirming the assay's validity. The absence of interaction between PtrTAC1‐1 and PtrLAZY1 suggests that their functions in regulating plant architecture are mediated through independent pathways.

### 2.7 RNA Sequencing Analysis of *TAC1‐CRISPR* Hybrid Poplar to Understand the Molecular Mechanism Behind the Upright Growth Architecture

**FIGURE 8:**
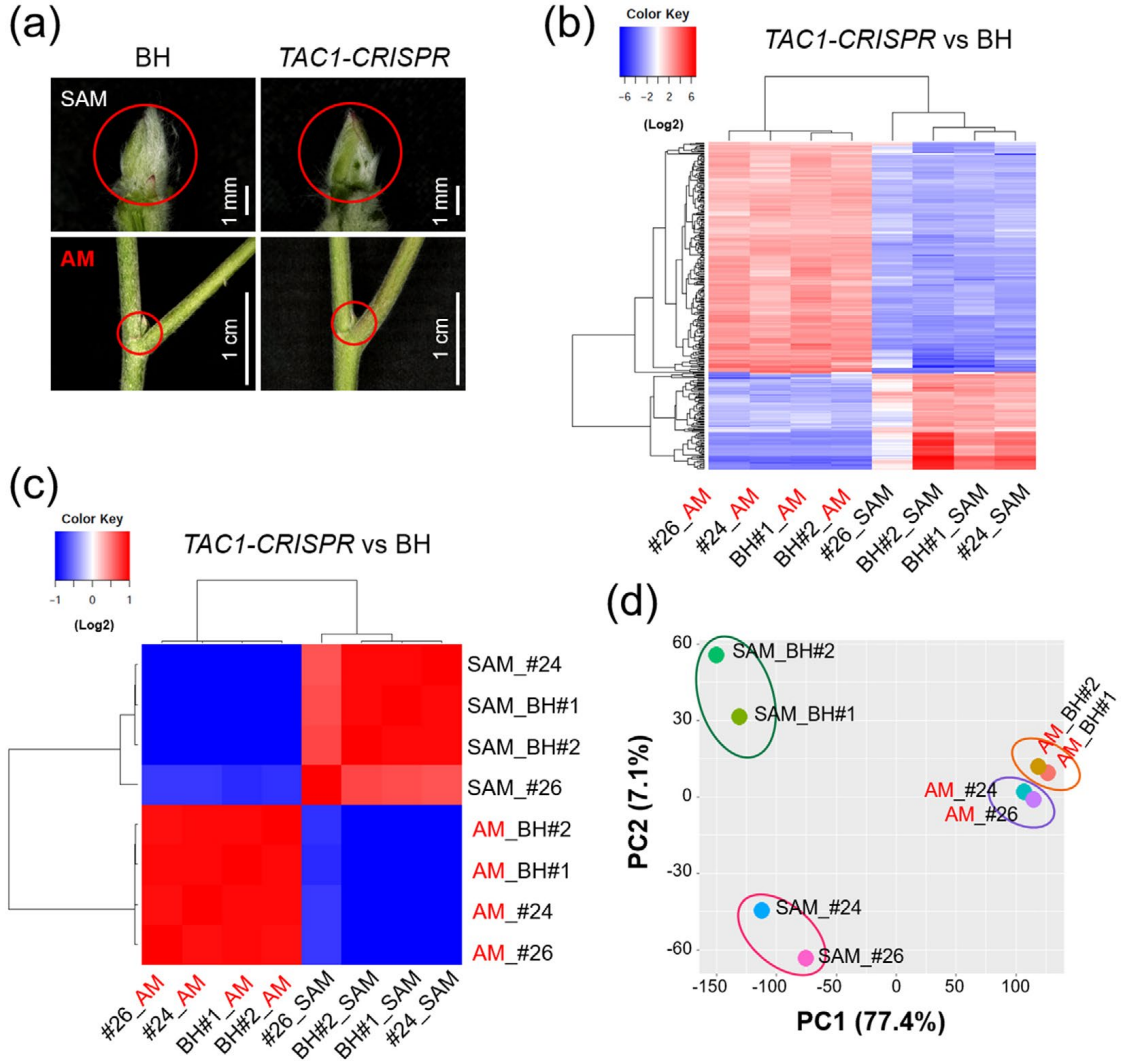
Transcriptome profiling of shoot apical meristem (SAM) and axillary meristem (AM) tissues in *TAC1‐CRISPR* and BH poplars. (a) Sampling scheme for RNA‐seq analysis. SAM tissues were dissected from the shoot apex after removing young leaves (top, red circles), and AM tissues were collected from the axils of the third to fifth nodes (bottom, red circles) in both BH and *TAC1‐CRISPR* hybrid poplar*s*. (b) Heatmap of hierarchical clustering based on differentially expressed genes (DEGs) between *TAC1‐CRISPR* and BH samples. DEGs were identified using the Trinity pipeline (analyze_diff_expr.pl) with log_2_ fold change values (*p* < 0.005). (c) Correlation matrix among samples, illustrating transcriptomic similarities and differences. Red indicates strong correlation; blue indicates weak correlation. (d) Principal component analysis (PCA) reveals distinct separation between BH and *TAC1‐CRISPR* samples. PC1 and PC2 account for 77.4% and 7.1% of the variance, respectively. PCA was performed using ggplot2 in R.

To investigate the molecular mechanisms underlying the upright growth architecture of *TAC1‐CRISPR* hybrid poplars, we performed a comparative transcriptome analysis on axillary meristem (AM) tissues, which are critical regulators of branching patterns, in both BH and *TAC1‐CRISPR* hybrid poplars. Additionally, shoot apical meristem (SAM) tissues were included as reference samples (Figure 8, see Section 4). Total RNA was extracted from eight tissue samples: four axillary meristem (AM) samples (two each from BH #1 and BH #2 and *TAC1‐CRISPR* lines #24 and #26) and four shoot apical meristem (SAM) samples (also two each from BH #1 and BH #2 and *TAC1‐CRISPR* lines #24 and #26) (Figure 8a). The RNA samples were subjected to RNA sequencing (RNA‐seq) analysis using the HiSeq 2000 platform (Illumina, San Diego, CA, USA). Although lines #24 and #26 do not include within‐line biological replicates, they represent two independent *TAC1‐CRISPR* knockout events with distinct editing patterns (Type‐1 and Type‐2, respectively). Both lines carried biallelic loss‐of‐function mutations across all *TAC1* homologues and displayed highly similar phenotypic outcomes, including upright growth habit and comparable physiological responses (Figures 3, 4, 5, 6, 7). Independent allelic knockouts that converge on the same phenotype are widely considered functional equivalents and are frequently used as biological replicates in plant functional genomics (Li et al. 2018; Wang et al. 2018). Accordingly, in this study, lines #24 and #26 were treated as independent biological replicates for the mutant condition, thereby ensuring that the observed transcriptomic changes reflect genuine consequences of TAC1 disruption rather than clonal artefacts.

To validate the reliability of the transcriptome datasets, we generated a correlation heatmap and matrix using the Trinity package (Grabherr et al. 2011; Haas et al. 2013) (Figure 8b,c). The samples segregated into two distinct clusters corresponding to the AM and SAM tissues, indicating robust tissue‐specific transcriptomic profiles. Within the AM group, transcriptomes of BH and *TAC1‐CRISPR* hybrid poplars (#24 and #26) formed separate subclusters, reflecting subtle but consistent transcriptomic differences between these genotypes. In contrast, the SAM group exhibited less clustering by genotype, suggesting fewer genotype‐dependent differences in transcriptomic profiles within this tissue type (Figure 8b,c). The PCA plot shows distinct separation between SAM and AM tissues along PC1, with further differentiation between BH and *TAC1‐CRISPR* hybrid poplar lines, indicating significant transcriptomic differences between tissue types and genotypes (Figure 8d).

To further verify the RNA‐seq data, we conducted quantitative real‐time PCR (qRT‐PCR) analysis of selected genes, including *PtrTAC1‐1*, *PtrTAC1‐2*, *PtrANT*, *PtrPME*, and 10 genes associated with secondary cell wall biosynthesis (Figure S8). The qRT‐PCR data closely matched the transcript per million (TPM) expression profiles from RNA‐seq, confirming the accuracy and reproducibility of the transcriptome analysis. In *TAC1‐CRISPR* hybrid poplars, both *PtrTAC1‐1* and *PtrTAC1‐2* were significantly downregulated compared to the BH controls (Figure S8), consistent with earlier findings (Figure 7).

### 2.8 Transcriptional Reprogramming in Axillary Meristems of *TAC1‐CRISPR* Hybrid Poplars

**FIGURE 9:**
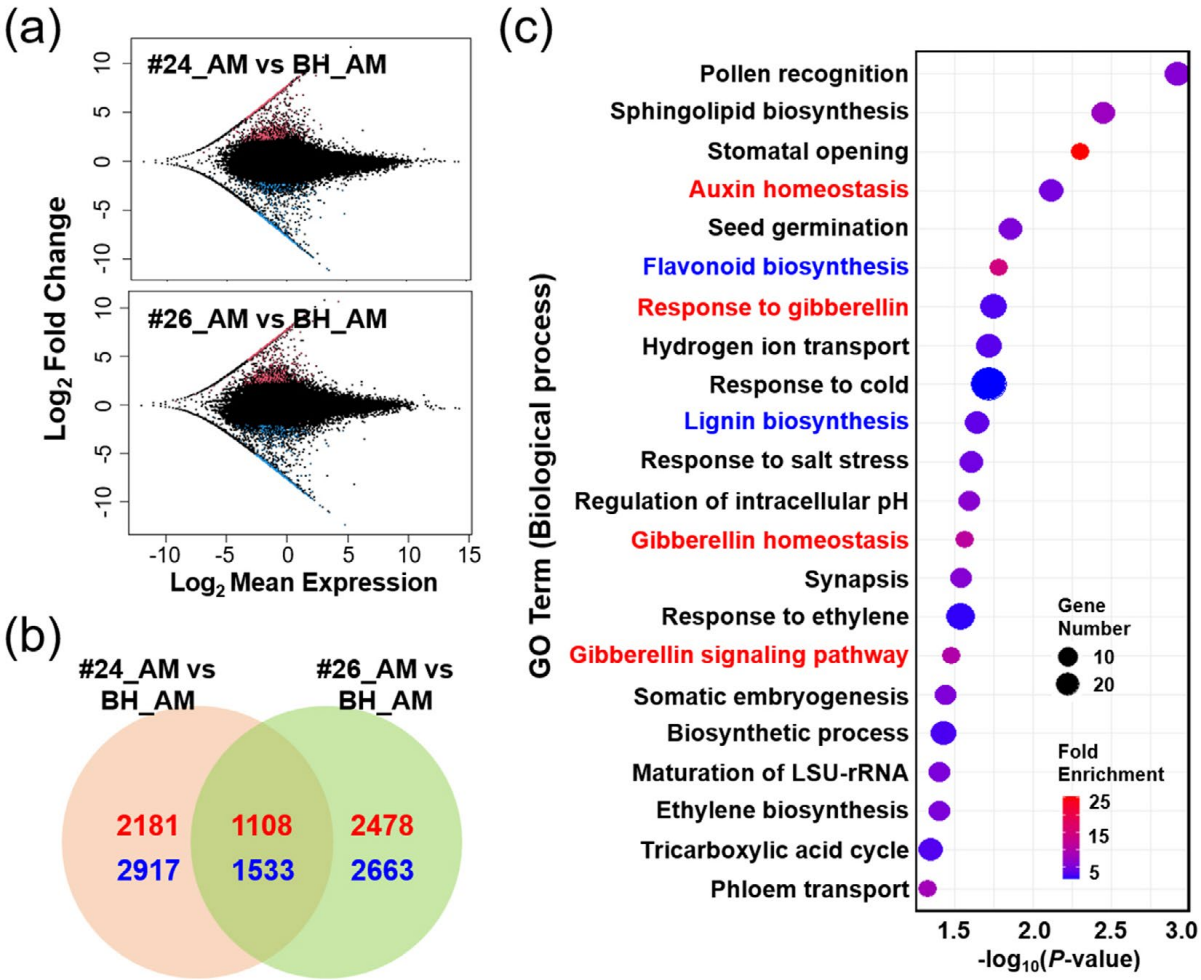
Differentially expressed genes (DEGs) and Gene Ontology (GO) enrichment in axillary meristems of *TAC1‐CRISPR* hybrid poplars. (a) MA plots displaying DEGs in *TAC1‐CRISPR* hybrid poplar (lines #24 and #26) relative to BH in axillary meristem (AM) tissues. Red and blue dots represent significantly upregulated and downregulated genes, respectively (adjusted *p* < 0.05). (b) Venn diagram showing the overlap of upregulated (red) and downregulated (blue) DEGs between the two *TAC1‐CRISPR* lines. (c) GO enrichment analysis of upregulated DEGs in *TAC1‐CRISPR* lines, categorised by biological processes. Only non‐overlapping GO terms (*p* ≤ 0.05), not shared with downregulated DEGs (Figure S9), are presented. Dot size corresponds to the number of genes, while colour intensity reflects fold enrichment, calculated as the ratio of gene frequency in the query set to that in the background.

We identified differentially expressed genes (DEGs) between the AM tissues of *TAC1‐CRISPR* hybrid poplar lines (#24_AM and #26_AM) and BH (BH_AM). MA plot analysis (Figure 9a) revealed 2641 shared DEGs, with 1108 upregulated and 1533 downregulated, across both #24_AM versus BH_AM and #26_AM versus BH_AM comparisons (Figure 9b). Gene Ontology (GO) analysis revealed significant enrichment of biological processes among the upregulated genes, particularly those associated with auxin homeostasis and gibberellin (GA) response (Figure 9c). These hormonal pathways are key regulators of branch angle and are likely modulated in *TAC1‐CRISPR* hybrid poplars to promote their upright growth architecture. Additionally, the upregulation of flavonoid and lignin biosynthesis pathways suggests enhanced structural support, which may further reinforce the columnar growth phenotype. In contrast, no enriched pathways relevant to upright growth architecture, to the best of our knowledge, were identified among the downregulated genes (Figure S9).

Several transcription factors (TFs) were upregulated in the AM tissues of *TAC1‐CRISPR* hybrid poplars (Table 1), including AUXIN RESPONSE FACTOR (ARF), which plays a central role in auxin‐mediated growth regulation. SCARECROW (SCR), a GRAS family member, is critical for gravity sensing and auxin redistribution (Di Laurenzio et al. 1996). Light‐responsive transcription factors such as B‐BOX proteins (BBX), ELONGATED HYPOCOTYL 5 (HY5) and PHYTOCHROME INTERACTING FACTOR 5 (PIF5) regulate photomorphogenic growth (Lau and Deng 2010; Leivar and Quail 2011). These findings highlight the molecular mechanisms through which *TAC1‐CRISPR* hybrid poplars achieve their altered architecture, emphasising the importance of hormonal regulation, structural reinforcement and efficient resource allocation. However, no significant TFs were identified among the downregulated genes (Table S1), warranting further investigation into potential repressive factors or post‐transcriptional regulation.

**TABLE 1:** Transcriptional regulators up‐regulated in the axillary meristem (AM) of *TAC1‐CRISPR* hybrid poplars compared to BH.

Contig ID^a^	AM_BH			AM_TAC1‐CRISPR		#24/BH^b^	#26/BH^c^	*Populus* ID (blastx)^d^	%^e^	*E*‐value^f^	Closest AGI (blastx)^g^	Gene description
	Rep #1	Rep #2	AVG	Line #24	Line #26							
DN107_c0_g1_i15:g.35956	0.0	0.0	0.0	4.8	3.3	12.2	11.7	Potri.017G028301.1	98.3	8E‐159	AT1G06040.1	BBX24. B‐BOX DOMAIN PROTEIN 24
DN5335_c0_g1_i2:g.96850	0.0	0.0	0.0	2.0	2.6	11.0	11.4	Potri.018G029500.1	96.3	1E‐79	AT5G11260.1	HY5, ELONGATED HYPOCOTYL 5
DN38586_c0_g1_i12:g.153398	0.0	0.0	0.0	1.4	1.1	10.4	10.1	Potri.001G015900.2	70.0	1E‐25	AT1G73730.1	SLIM1, Sulfur Limitation1
DN16137_c0_g1_i1:g.69606	0.0	0.0	0.0	1.0	1.0	9.9	9.9	Potri.001G257600.1	96.1	1E‐82	AT5G12840.4	NF‐YA1, Nuclear factor Y, subunit A1
DN1625_c0_g1_i23:g.170753	0.0	0.0	0.0	0.8	1.0	9.7	9.9	Potri.004G074300.1	96.3	3E‐72	AT5G17300.1	RVE1, REVEILLE 1
DN338_c0_g1_i12:g.109925	0.0	0.0	0.0	0.3	0.9	8.1	9.9	Potri.005G075100.1	95.8	0	AT2G22300.2	SR1, SIGNAL RESPONSIVE1
DN6619_c0_g1_i17:g.64937	0.0	0.0	0.0	0.5	0.8	8.8	9.6	Potri.002G188700.6	94.1	0	AT2G47070.1	SPL1, SQUAMOSA PROMOTER BINDING PROTEIN‐LIKE 1
DN15736_c0_g1_i9:g.112619	0.0	0.0	0.0	1.4	0.7	10.4	9.5	Potri.002G154000.1	94.8	3E‐105	AT1G33060.1	ANAC014, NAC domain protein 14
DN28698_c0_g1_i1:g.53149	0.0	0.0	0.0	0.5	0.7	9.0	9.5	Potri.001G219100.1	93.8	2E‐73	AT5G04760.1	DIV2, DIVARICATA2
DN3696_c0_g2_i4:g.15795	0.0	0.0	0.0	2.0	0.7	10.9	9.5	Potri.013G054000.1	88.2	0	AT3G04070.1	ANAC047, NAC domain protein 47
DN33784_c0_g1_i12:g.102230	0.0	0.0	0.0	0.4	0.6	8.8	9.3	Potri.019G036100.1	90.2	3E‐150	AT5G19790.1	ERF/AP2 transcription factor
DN108550_c0_g1_i1:g.134	0.0	0.0	0.0	0.3	0.6	8.2	9.3	Potri.016G024000.1	93.0	7E‐63	AT3G56850.1	ABA‐responsive bZIP protein
DN36663_c0_g1_i8:g.46164	0.0	0.0	0.0	0.3	0.6	8.0	9.3	Potri.008G129500.2	94.3	3E‐134	AT2G01430.1	HB17, HD‐Zip transcription factor
DN14713_c0_g1_i30:g.139042	0.0	0.0	0.0	0.5	0.6	8.9	9.1	Potri.016G027750.1	63.4	9E‐24	AT1G66350.1	RGA‐LIKE 1, DELLA protein
DN31984_c0_g1_i2:g.105143	0.0	0.0	0.0	0.2	0.4	7.8	8.6	Potri.009G075500.2	95.1	0	AT3G54220.1	SCR, GRAS transcription factor
DN12452_c1_g1_i11:g.24348	0.0	0.0	0.0	1.5	0.4	10.5	8.6	Potri.011G023600.1	100.0	2E‐88	AT1G79430.2	APL, ALTERED PHLOEM DEVELOPMENT
DN2626_c0_g1_i15:g.30939	0.0	0.0	0.0	1.6	0.3	10.6	8.4	Potri.003G001000.2	95.5	0	AT1G59750.4	ARF1, AUXIN RESPONSE FACTOR 1
DN27369_c0_g1_i2:g.25251	0.0	0.0	0.0	0.4	0.3	8.7	8.4	Potri.004G155400.3	96.1	9E‐51	AT1G75250.1	RAD‐LIKE 6 protein
DN64497_c0_g1_i1:g.113938	0.0	0.0	0.0	0.2	0.3	7.7	8.2	Potri.001G326000.3	100.0	4E‐57	AT2G01570.1	RGA, DELLA protein
DN2252_c0_g1_i17:g.29117	0.0	0.0	0.0	0.8	2.0	8.3	9.6	Potri.007G007100.1	92.2	0	AT2G22840.1	GRF1, growth‐regulating factor 1
DN518_c0_g1_i9:g.147623	0.0	0.0	0.0	2.4	3.0	6.9	7.3	Potri.005G207200.14	90.4	0	AT3G59060.1	PIF5, PHYTOCHROME‐INTERACTING FACTOR 5
DN15285_c0_g1_i10:g.106932	0.0	0.1	0.1	0.7	1.3	3.5	4.4	Potri.005G053200.1	95.4	9E‐57	AT5G28770.3	bZIP63, Basic leucine zipper protein 63
DN14076_c0_g5_i1:g.93184	0.2	0.0	0.1	0.6	1.6	2.7	4.2	Potri.001G058800.1	95.7	7E‐52	AT5G13080.1	WRKY75, WRKY transcription factor
DN7852_c0_g1_i1:g.163620	1.3	0.0	0.6	6.0	9.9	3.2	3.9	Potri.003G169700.2	94.3	1E‐119	AT1G26310.1	AGL10, AGAMOUS‐LIKE 10
DN23645_c1_g1_i7:g.22767	0.0	0.1	0.0	0.2	0.5	2.9	3.8	Potri.013G098550.2	93.2	2E‐136	AT4G12020.1	WRKY19, WRKY transcription factor
DN27156_c0_g1_i13:g.82347	0.0	0.3	0.1	0.9	1.6	2.7	3.4	Potri.019G091900.1	95.4	0	AT3G58120.1	Basic leucine zipper protein 61
DN38169_c0_g1_i1:g.165194	0.1	0.0	0.1	0.3	0.5	2.7	3.4	Potri.016G027750.1	98.4	6E‐130	AT1G14920.1	GAI, DELLA protein
DN809_c0_g1_i5:g.61315	0.3	0.0	0.2	1.4	1.4	3.1	3.0	Potri.010G136100.1	92.4	2E‐118	AT1G68920.3	bHLH49, basic helix–loop–helix protein
DN5809_c0_g1_i8:g.49713	0.1	0.0	0.1	0.7	0.5	3.4	3.0	Potri.005G117600.1	89.8	0	AT5G66320.2	GATA transcription factor 5
DN22687_c0_g1_i7:g.55607	0.1	0.0	0.1	0.3	0.4	2.6	2.9	Potri.015G099200.1	80.9	1E‐97	AT5G13080.1	WRKY75, WRKY transcription factor

^a^ Contig ID obtained from Trinity‐v2.15.2.

^b^ Log_2_ fold change (Log_2_FC), calculated by Log_2_((TPM of #24 AM + 0.001)/(TPM of BH AM + 0.001)).

^c^ Log_2_FC calculated as described in ‘^b^’.

^d^ Poplar gene ID from *P. trichocarpa* v4.1 (Phytozome v13).

^e^ % Identity from blastX against *P. trichocarpa* v4.1.

^f^ Statistical significance from blastX against *P. trichocarpa* v4.1.

^g^ *Arabidopsis* gene ID from TAIR10.

### 2.9 Hormonal and Photomorphogenic Rewiring Shapes Upright Growth in *TAC1‐CRISPR* Hybrid Poplars

**FIGURE 10:**
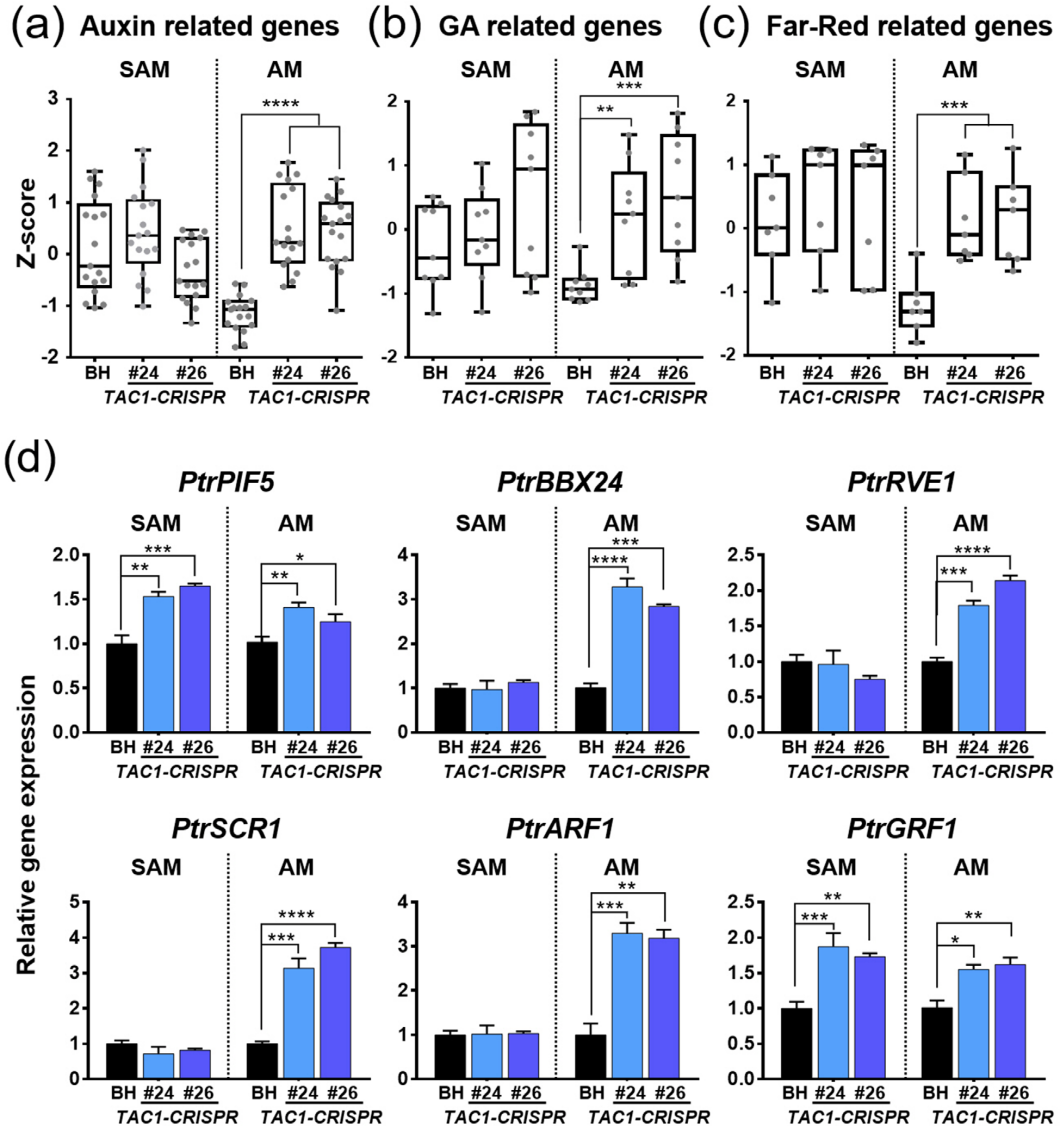
Coordinated hormonal and light signalling reprogramming underlies upright architecture in *TAC1‐CRISPR* hybrid poplars. (a–c) Boxplots showing normalised gene expression (*Z*‐scores) for genes involved in (a) auxin biosynthesis and signalling, (b) GA pathways, and (c) far‐red light responses in SAM and AM tissues of BH and *TAC1‐CRISPR* hybrid poplar lines (#24 and #26). Gene sets are listed in Table S2. (d) Validation of RNA‐seq data by qRT‐PCR for selected genes related to light and auxin signalling. Genes shown include light‐responsive factors (*PtrPIF5*, *PtrBBX24*, *PtrRVE1*), gravity‐associated transcription factor (*PtrSCR1*), and auxin signalling regulators (*PtrARF1*, *PtrGRF1*). Relative expression is shown for SAM and AM tissues across genotypes. Statistical significance was calculated using Student's *t*‐test: *p < 0.1, **p < 0.01, ***p < 0.001, ****p < 0.0001.

Next, we examined the differential regulation of auxin, GA, and photomorphogenesis‐related genes in the AM tissues. Normalised expression (*Z*‐scores) of these genes was analysed and displayed in boxplots for SAM and AM tissues of BH and *TAC1‐CRISPR* hybrid poplars (Figure 10a–c; Table S2). In the AM tissues of *TAC1‐CRISPR* hybrid poplars, auxin‐related genes, including those involved in biosynthesis and transport, and GA signalling genes were significantly upregulated compared to BH (Figure 10a,b), indicating enhanced hormonal signalling that may contribute to the upright growth phenotype. Additionally, the differential expression of far‐red light‐responsive genes (Figure 10c) suggests that photomorphogenic pathways are also engaged in modulating branch orientation and shoot architecture.

To validate these findings, we conducted qRT‐PCR analysis of key transcriptional regulators highlighted in the transcriptome data, including light and circadian signalling components (*PtrPIF5*, *PtrBBX24*, *PtrRVE1*), the auxin response factor (*PtrARF1*), the growth regulator (*PtrGRF1*), and the gravity‐sensing gene (*PtrSCR1*) (Figure 10d). *PtrBBX24*, *PtrRVE1*, *PtrSCR1* and *PtrARF1* were significantly upregulated specifically in AM tissues of *TAC1‐CRISPR* lines, but not in SAM, suggesting that these genes respond locally to the loss of *TAC1*. In contrast, *PtrPIF5* and *PtrGRF1* were upregulated in both SAM and AM, indicating broader transcriptional reprogramming. Together, these results suggest that TAC1 influences shoot architecture through spatially distinct regulation of transcription factors integrating hormonal, light and gravity signals.

**FIGURE 11:**
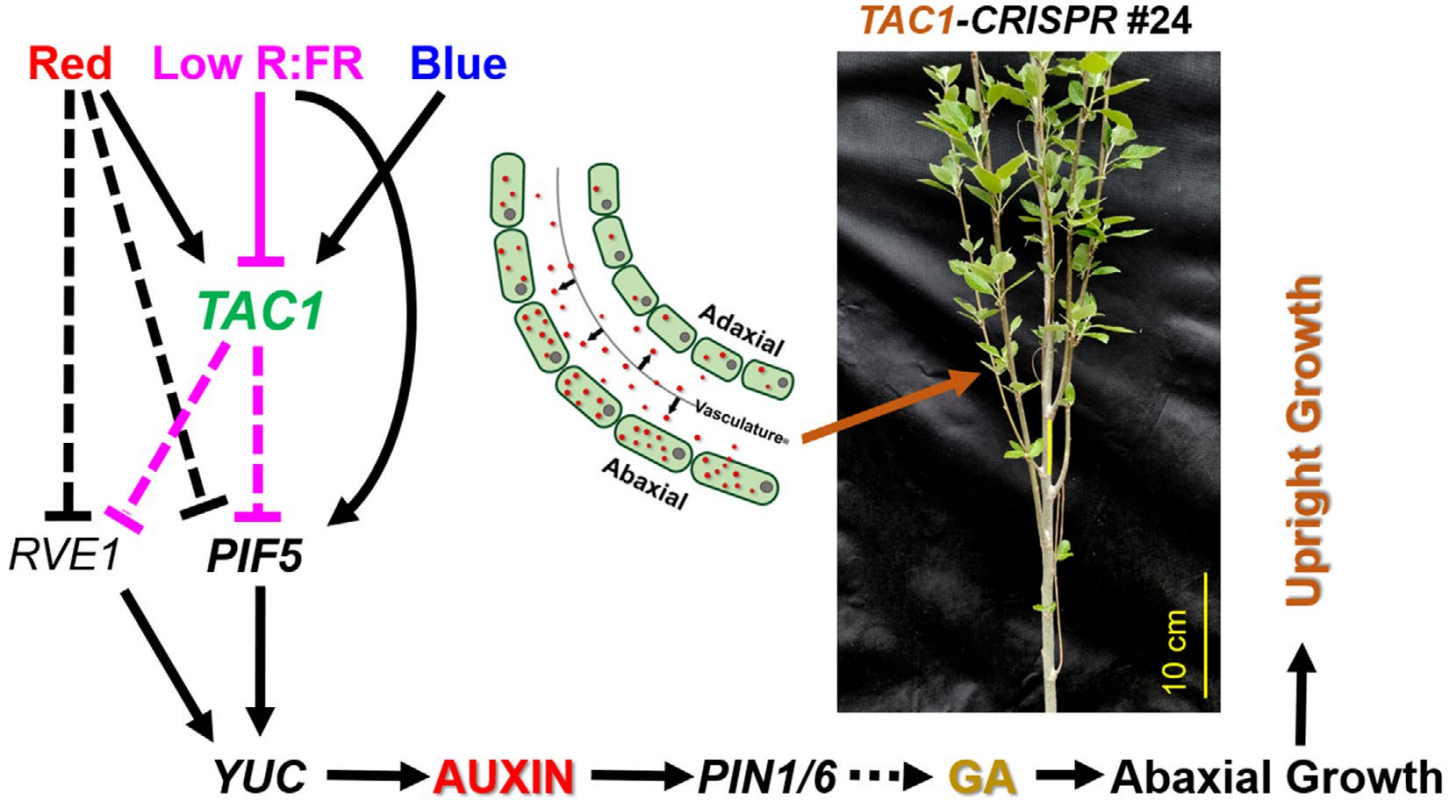
Proposed model for TAC1‐mediated regulation of plant architecture. Loss of *TAC1* in *TAC1‐CRISPR* lines results in the derepression of auxin biosynthesis and transport genes, redirecting auxin flow toward the abaxial side of axillary meristems (AMs). This auxin redistribution, together with enhanced gibberellin biosynthesis, promotes asymmetric cell elongation, leading to steeper branch angles and an upright growth habit. The image of *TAC1‐CRISPR* #24 exemplifies this architectural phenotype.

Based on these findings, we propose a working model (Figure 11) illustrating how *TAC1* disruption reprograms molecular pathways to promote upright growth. TAC1 appears to coordinate signalling among light, auxin and GA pathways. In its absence, increased expression of light‐responsive transcription factors (e.g., *PIF5* and *RVE1*) and auxin‐related genes (e.g., *YUC*, *PIN1*, *PIN6*) likely shifts auxin gradients toward the abaxial side of the AM, resulting in steeper branch angles. Simultaneously, upregulation of GA biosynthesis supports cell elongation, further reinforcing vertical shoot architecture. The upright phenotype observed in *TAC1‐CRISPR* line #24 (Figure 11) visually reflects these molecular changes. Together, these results highlight the integrative role of TAC1 in orchestrating hormonal and environmental signalling networks to regulate branch architecture and promote upright growth.

## 3 Discussion

Lombardy poplar ( *P. nigra* var. *italica* ) is characterised by its columnar shape and upright branching, traits valued for landscaping and afforestation (Zsuffa 1974). While the molecular basis of this architecture was previously unknown, it was hypothesized to involve mutations in a poplar *TAC1* ortholog. In this study, we identified a nonsense mutation in the third exon of *PnTAC1‐1*, introducing a premature stop codon and likely resulting in loss of function, whereas *PnTAC1‐2* remained intact (Figure 1; Figure S2). This finding aligns with previous reports showing that loss‐of‐function mutations in *TAC1* orthologs lead to upright branching in various woody species, including pillar peach (Dardick et al. 2013; Hollender, Waite, et al. 2018), hybrid poplar (Fladung 2021), sweet orange (Dutt et al. 2022) and cotton (Kangben et al. 2024). Consistent with prior evidence that disruption of a single *TAC1* homologue (e.g., *TAC‐14*) can induce columnar growth (Fladung 2021), our results strongly support the conclusion that the *PnTAC1‐1* mutation is the primary genetic cause of the Lombardy poplar's columnar phenotype. These findings reinforce the role of TAC1 as a key regulator of tree architecture and its potential utility in breeding programs targeting improved growth form, planting density and biomass productivity.

### 3.1 *TAC1‐CRISPR* Hybrid Poplars Exhibit an Upright Growth Habit With Reduced Petiole Angles, Phenotypically Analogous to Lombardy Poplar

To confirm that the nonsense mutation in *PnTAC1‐1* is responsible for the columnar phenotype of Lombardy poplar, a genetic complementation experiment would be ideal. However, no genetic transformation system currently exists for Lombardy poplar. As an alternative approach to assess the functional role of TAC1 in tree architecture, we employed CRISPR/Cas9 genome editing to disrupt all four *TAC1* homologue alleles (*PaTAC1‐1*, *PaTAC1‐2*, *PgTAC1‐1* and *PgTAC1‐2*) in hybrid poplar (Figure 2). The resulting *TAC1‐CRISPR* hybrid poplars displayed a stable columnar growth phenotype, with significantly narrower petiole and branch angles compared to wild‐type BH, closely resembling Lombardy poplar (Figures 3 and 4). This architectural phenotype was consistently observed under both controlled greenhouse conditions and in long‐term LMO field trials (Figure 4). Field evaluations were conducted over 2 years under fluctuating environmental conditions, including winter, using a randomised grid design with 30 replicates per genotype for robust statistical analysis (Figure 4). Consistent results were also obtained from an independent set of CRISPR‐TAC1 hybrid poplars generated with a CRISPR/Cas9 geminiviral replicon vector, which displayed the same columnar phenotype (Figure S7). Despite the drastic change in branch architecture, key growth parameters such as stem height and diameter remained comparable to wild‐type trees (Figure 4d,e), indicating that *TAC1* knockout effectively modifies tree form without compromising growth performance.

Although our approach targeted all *TAC1* homologues to ensure complete functional loss and a stable architectural phenotype for long‐term field evaluation, we recognise that *TAC‐14* in *Populus* × *canescens*—orthologous to *PnTAC1‐1* in *P. nigra* —was shown by Fladung (2021) to be sufficient on its own to induce a columnar phenotype. Generating independent knockouts of *TAC1‐1* and *TAC1‐2* in the same genetic background would help determine the extent of functional redundancy or specialisation between these homologues. Such work would also clarify whether *TAC1‐2* contributes to architecture in certain backgrounds despite the strong effect of *TAC1‐1* disruption. Future studies dissecting these homologue‐specific roles could refine molecular breeding strategies for tailoring tree form.

### 3.2 Asymmetric Cell Elongation Drives Upright Growth in *TAC1‐CRISPR* Hybrid Poplars

Anatomical analysis of *TAC1‐CRISPR* hybrid poplars revealed that their upright growth habit primarily results from asymmetric cell elongation in the lower petiole region rather than increased cell division (Figure 5). Such asymmetric cell elongation is a conserved mechanism influencing plant architecture and gravitropic responses across species. For instance, in *Arabidopsis*, the Shade Avoidance Syndrome (SAS) triggers auxin‐mediated asymmetric cell elongation in hypocotyls, promoting upward growth under low red‐to‐far‐red light conditions (Ma and Li 2019). Similarly, gravitropic bending in *Arabidopsis* roots occurs through differential elongation of cells, allowing roots to curve downward (Rath et al. 2020). Comparable mechanisms are seen in legumes like soybean and Mimosa, where asymmetric elongation in the pulvinus drives leaf movements responding to gravity and light (Zeng et al. 2024). Furthermore, phototropic and gravitropic responses involve auxin‐driven asymmetric cell elongation to control shoot orientation in *Populus tremula* × *Populus alba* (Žádníková et al. 2015). Thus, the localised elongation observed in *TAC1‐CRISPR* hybrid poplar petioles aligns well with this widely conserved developmental strategy, highlighting the critical role of targeted cell expansion in shaping whole‐plant architecture.

Additionally, *TAC1‐CRISPR* hybrid poplars exhibited an enhanced gravitropic response, with faster shoot reorientation and recovery after a 90° tilt, indicating heightened sensitivity to gravitational signals (Figure 6). Notably, this enhanced gravitropic response occurred without alterations in *PtrLAZY1* expression, a gene traditionally linked to gravitropism and branch angle control, and in the absence of detectable protein interactions between PtrTAC1 and PtrLAZY1 (Figure 7). These observations suggest that TAC1 and LAZY1 operate independently yet possibly through complementary regulatory mechanisms. Interestingly, contrasting findings in citrus showed increased *CsLAZY1* expression upon *CsTAC1* silencing (Dutt et al. 2022), highlighting possible species‐specific regulatory differences. Future research should explore alternative pathways and additional IGT gene family members that might cooperate with or compensate for TAC1 function. Genetic analyses, such as generating double mutants or performing genetic interaction assays, would provide valuable insights into the molecular networks controlling gravitropism and plant architecture.

### 3.3 Transcriptomic Insights Into Hormonal and Photomorphogenic Control of Upright Architecture in *TAC1‐CRISPR* Hybrid Poplars

Our transcriptome analyses provided important insights into the molecular mechanisms underlying the upright growth phenotype of *TAC1‐CRISPR* hybrid poplars (Figures 8, 9, 10). Expression levels of *PtrTAC1‐1* and *PtrTAC1‐2* were substantially higher in AM than in SAM tissues of BH poplars (Figure S10), supporting their potential role in branch angle regulation. This observation aligns with previous reports showing elevated *TAC1* expression in actively growing vegetative buds despite low expression in the meristem (Xu et al. 2017). The observed upregulation of genes involved in auxin biosynthesis, transport, and GA signalling indicates a complex hormonal crosstalk that significantly influences plant architecture. This observation is consistent with previous findings in citrus, where CRISPR‐mediated disruption of *CsTAC1* altered endogenous hormone levels (Dutt et al. 2022). Similarly, transcriptomic profiling of pillar peach trees indicated auxin‐related gene expression changes in response to *TAC1* loss (Hollender, Waite, et al. 2018). Furthermore, the differential expression of far‐red light‐responsive transcription factors such as *HY5* and *PIF5* suggests that photomorphogenic signalling interacts with hormonal pathways to regulate branch angles. Supporting this, recent evidence from peach (Waite et al. 2025) shows that PpeTAC1 protein interacts with chloroplast‐ and light‐related proteins, highlighting its role in integrating light cues to control lateral organ orientation. Collectively, these findings support a model in which TAC1 serves as a central integrator of environmental signals and hormonal pathways, orchestrating plant architectural development.

Several transcription factors upregulated in AM tissues of *TAC1‐CRISPR* hybrid poplars (Table 1) provide additional clues to the molecular basis of upright architecture. Auxin response factors (ARFs) and HY5 are pivotal in auxin‐driven growth regulation and branch orientation (Cluis et al. 2004; Carey and Krogan 2017). Upregulation of *SCARECROW* (*SCR*), a GRAS family transcription factor, reinforces gravitropic sensitivity through enhanced auxin redistribution (Sharma et al. 2022). The elevated expression of *BBX* transcription factors (e.g., *BBX24*) further suggests that *TAC1* disruption triggers photomorphogenic signalling pathways aimed at optimising branch positioning for improved light capture efficiency (Cao et al. 2023). BBX24's interaction with regulators such as HY5 and COP1 (Jiang et al. 2012; Gangappa et al. 2013) indicates that *TAC1* knockout impacts shoot architecture through an intricate balance of hormonal, photomorphogenic and gravitropic signals, presenting valuable targets for future tree breeding strategies to optimise plant architecture.

In conclusion, our findings confirm TAC1 as a key genetic regulator of tree architecture, mediating branch orientation and gravitropic responses through the coordination of hormonal and photomorphogenic signalling pathways. The development of *TAC1‐CRISPR* hybrid poplars exhibiting stable upright growth highlights the practical potential of gene editing technologies for optimising tree form. Such upright architectures facilitate increased planting densities, improved land‐use efficiency, and simplified harvesting, potentially reducing operational costs and enhancing biomass production (Mantilla‐Perez and Salas Fernandez 2017; Fladung 2021; Dutt et al. 2022; Kangben et al. 2024). Future studies should focus on evaluating long‐term field performance, biomass yield and wood quality in *TAC1*‐modified trees. Comprehensive ecological and environmental assessments will also be essential prior to the broad implementation of genetically modified woody plants in sustainable forestry and biomass applications.

## 4 Materials and Methods

### 4.1 Plant Materials and Growth Conditions

Two male Lombardy poplar trees ( *Populus nigra* var. *italica* ) were obtained from the Forest Bio‐resources Department, National Institute of Forest Science (NIFoS), Republic of Korea. Hybrid poplars ( *P. alba* × *P. glandulosa* , clone BH) were used as wild‐type controls and for transgenic experiments. In vitro cultures were maintained on half‐strength Murashige and Skoog (MS) medium (M0222; Duchefa Biochemie, Haarlem, the Netherlands) supplemented with 3% sucrose and 0.8% phyto agar (P1003; Duchefa Biochemie, Haarlem, the Netherlands). Plants were acclimated to soil and grown either in a controlled growth room (16‐h light, 150 μmol m^−2^ s^−1^, 24°C) or in an LMO field at NIFoS (37.2° N, 126.9° E). The field trial included five *TAC1‐CRISPR* knockout lines (#8, #24, #25, #26 and #27; Type‐1 and Type‐2 events) and the wild‐type BH control. Thirty biological replicates per genotype were planted in a randomised grid layout to minimise environmental variation and enable robust genotype comparisons (Mead 1988; Piepho et al. 2003).

### 4.2 Measurement of Poplar Growth

Stem height was measured using a ruler or measuring pole from the top of the plant to soil level, and stem diameter was measured using digital callipers (Mitutoyo, Kawasaki, Japan) at 5 cm above soil level. Petiole or branch angles were measured at the 4th to 10th petioles from the top in potted plants, or at three nodes located 1 m above the ground in field‐grown plants. Three biological replicates per line were analysed.

### 4.3 Vector Construction and Plant Transformation

Using full‐length cDNA sequences of *TAC1* genes (i.e., *PaTAC1‐1*, *PgTAC1‐2*, *PaTAC1‐2* and *PgTAC1‐2*) from our hybrid poplar, single‐guide RNAs (sgRNAs) targeting these genes were designed using Cas‐Designer in CRISPR RGEN Tools (http://www.rgenome.net/cas‐designer/20210817), with the *Populus alba* × *P. tremula* var. *glandulosa* (Poplar 84K) genome (GenBank accession: GCA_014332715.1) as the reference (Qiu et al. 2019). Candidate targets were filtered for low predicted mismatches and high out‐of‐frame scores, with only those lacking predicted off‐target sites containing fewer than three mismatches in the seed region retained. Two sgRNAs (sg1 and sg2) were ultimately selected to knock out both TAC1‐1 and TAC1‐2, with a guide length of 20 bp excluding the PAM sequence (Figure S3).

To make *TAC1‐CRISPR* hybrid poplars, we used the pHSE401 vector for *TAC1‐CRISPR* vector construction. In brief, the U6‐promoter and U6‐terminator were amplified using the pHSE401 plasmid as a template to include two sgRNA sequences into a single vector. Overlapping PCR was used to obtain the target sequence of two sgRNAs, U6‐promoter, and other essential elements, approximately 808 bp. The amplified target sequence was purified and digested with *Bsa*I (ThermoFisher Scientific, MA, USA) together with the pHSE401 vector, and the digested products were ligated using T4 DNA ligase (Promega, WI, USA). The ligation products were transformed into *E. coli* cells, and the recombinant clones were selected by colony PCR.

TAC1‐CRISPR replicon constructs were generated using the pLSL.R.Ly backbone (Vu et al. 2020) to produce pPLID4, which incorporated long and short intergenic regions (LIR and SIR), and a lycopene marker from pLSLR (Čermák et al. 2015). Editing cassettes included a poplar ANT1‐based RNAi inducer (PtRTFi), a plant codon‐optimised Cas9 (35S‐pcoCas9I‐t35S), two TAC1‐targeting gRNAs, a selection marker (pNOS‐NptII‐tOCS; Addgene #51144), and an end‐linker (pEndL4; Addgene #48019). Assembly was performed by Golden Gate cloning (Engler et al. 2014; Kim et al. 2025). Recombinant clones were screened by colour selection and verified by Sanger sequencing.

Vector constructs were then introduced into *Agrobacterium tumefaciens* strain C58, which was used to transform poplar by the stem or petiole segment transformation–regeneration method (Horsch et al. 1985; Choi et al. 2005; Bae et al. 2021). Briefly, stem or petiole segments from in vitro‐grown shoots were excised and cut into ~1 cm segments, which were then incubated with *Agrobacterium tumefaciens* strain C58 harbouring the CRISPR/Cas9 binary vector for 10 min. After co‐cultivation for 3 days on hormone‐supplemented callus induction medium under dark conditions, the explants were transferred to shoot induction medium containing antibiotics for selection. Emerging shoots were excised and transferred to rooting medium. All constructs used in this study were verified by DNA sequencing (Macrogen, Seoul, South Korea).

### 4.4 Genotyping of Mutated Sequences

Genomic DNA was extracted from leaves of Lombardy poplar and transgenic hybrid poplars using a modified cetyltrimethylammonium bromide (CTAB) method (Yu et al. 2019). In brief, leaf tissues were ground into a fine powder using liquid nitrogen and mixed with CTAB buffer (2% CTAB, 100 mM Tris–HCl [pH 8.0], 25 mM EDTA [pH 8.0], 2 M NaCl), followed by chloroform extraction. Isopropanol was added to the mixture to isolate DNA. For Lombardy poplar, TAC1 target regions were amplified by gDNA‐PCR with specific primers (Table S3) using 10 ng/μL DNA as a template. PCR products were resolved on 1% agarose gels, purified (GeneAll Expin GEL SV), and sequenced by Sanger sequencing (Macrogen, https://www.macrogen.com/). For *TAC1‐CRISPR* poplars, a ~500 bp region spanning the gRNA site was amplified, re‐amplified with adapter and index primers to generate ~200 bp amplicons, purified, and sequenced on the Illumina MiniSeq platform (SY‐420‐1001; KAIST Bio Core Center, https://biocore.kaist.ac.kr/). Mutations were analysed using Cas‐Analyser (CRISPR RGEN Tools, http://www.rgenome.net/).

### 4.5 Histological Analysis of Petiole Tissues

Petioles from the 13th to 14th nodes of 3‐month‐old soil‐grown poplars were collected for histological analysis. Microtome sectioning and toluidine blue O (TBO) staining were performed as previously described (Chen et al. 2016; Jeon et al. 2016). Briefly, samples were fixed in FAA solution (50% ethanol, 10% formaldehyde, 5% glacial acetic acid) under vacuum for 15 min, dehydrated through a graded ethanol series, and cleared with ethanol:xylene substitute mixtures. After paraffin embedding, 8 μm sections were prepared using a rotary microtome (RM2125 RTS; Leica). Paraffin was removed with two washes in 100% xylene, followed by rehydration through a graded ethanol series. Sections were stained with 0.05% TBO for 1 min. Images were captured using a microscope (CHB‐213; Olympus, Tokyo, Japan) and analysed with ImageJ software (NIH, http://www.nih.gov/).

### 4.6 Yeast Two Hybrid Assay

The yeast two‐hybrid assay was conducted following the method described by Bae et al. (2020). Briefly, the full‐length *PtrTAC1‐1* cDNA was cloned into the pGBKT7 bait vector (Addgene #61703), fused in‐frame with the GAL4 DNA‐binding domain, and selected using tryptophan (Trp) dropout media. The *PtrLAZY1* cDNA fragments were cloned into the pGADT7 prey vector (Addgene #61702), fused in‐frame with the GAL4 activation domain, and selected on leucine (Leu) dropout media. The combination of *AtSTM*/AD and *PtrTALE12ΔC*/BD served as a positive control, while empty pGBKT7 and pGADT7 vectors were used as negative controls. All constructs were transformed into the *Saccharomyces cerevisiae* strain AH109. Protein–protein interactions were assessed on selective media: SD‐W (−Trp), SD‐LW (−Leu, −Trp) and SD‐AHLW (−Ade, –His, −Leu, −Trp).

### 4.7 RNA Extraction and Quantitative Real‐Time PCR (qRT‐PCR) Analysis

Total RNA was extracted from young leaves of field‐grown poplars. For RNA‐seq analysis, shoot apical meristem (SAM) and axillary meristem (AM) tissues were harvested from three 2‐year‐old poplar trees (Figure 8a). The collected tissues were immediately frozen in liquid nitrogen and stored at −80°C. Total RNA was extracted using the RNeasy Plant Mini Kit (Qiagen, Hilden, Germany) according to the manufacturer's instructions. The RNA integrity was assessed using the Agilent 2100 Bioanalyzer, and only samples with a RIN value of 8.0 or higher were used for subsequent analysis.

Quantitative real‐time PCR was performed on a CFX96 Real‐Time System (Bio‐Rad) using SYBR Green PCR Master Mix (Bio‐Rad, Hercules, CA, USA). One microgram of total RNA was reverse transcribed into cDNA using the SuperScript III Reverse Transcriptase (Invitrogen, Carlsbad, CA, USA). Poplar *ACTIN2* (Potri.019G010400) was used as the internal reference gene, and relative gene expression levels were calculated using the 2^−∆∆CT^ method (Livak and Schmittgen 2001). All primers were designed using Primer3Web (version 4.1.0; primer3.ut.ee) and are listed in Table S3.

### 4.8 RNA‐Seq Data Processing and Analysis

Raw paired‐end FASTQ files were processed using Trimmomatic PE (v0.39) for adapter trimming and quality filtering (parameters: ILLUMINACLIP: <fastaWithAdapters>:2:30:10:2 SLIDINGWINDOW:5:20 LEADING:20 TRAILING:20 MINLEN:50), followed by Prinseq‐lite (v0.20.4) to remove low‐quality and short read sequences. Genome‐guided transcriptome assembly was performed via Trinity (v2.15.2) within a Docker environment. The assembled transcripts were subjected to open reading frame (ORF) prediction using TransDecoder (v5.5.0), and coding sequences were identified based on BLASTp results against the UniProt‐SwissProt database and Pfam domain searches using HMMER (v3.3.2). TransDecoder retained ORFs supported by both BLASTp and Pfam annotations. Transcript abundance was quantified using kallisto through Trinity's align_and_estimate_abundance.pl script. Transcripts per million (TPM) across all samples were merged into a unified expression matrix using abundance_estimates_to_matrix.pl. Contig sequences were functionally annotated using BLASTx (v2.12.0+) against the *P. trichocarpa* v4.1 and *A. thaliana* TAIR10 protein databases with an *E*‐value cutoff of 1E‐5. Data analysis was conducted using Excel for data processing, GraphPad Prism 6 and R (v4.4.1) for statistical analyses and data visualisation of expression patterns and heatmap.

### 4.9 Differentially Expressed Genes (DEGs) Functional Analysis

DEGs were classified using strict criteria. Upregulated DEGs were defined as those showing ≥ 5‐fold higher TPM values in both TAC1‐CRISPR lines (#24 and #26) compared to average BH TPM, while downregulated DEGs required ≥ 5‐fold lower TPM values in both lines for contigs expressed in BH (average BH TPM > 0). For contigs with BH TPM = 0, upregulated DEGs were defined as those with TPM values in both *TAC1‐CRISPR* lines exceeding twice the average *TAC1‐CRISPR* #24 and #26 TPM. A total of 1108 upregulated DEGs (36 transcription factors [TFs]) and 1533 downregulated DEGs (50 TFs) were identified. Gene Ontology (GO) enrichment analysis of biological processes was performed for DEGs using The Database for Annotation, Visualisation and Integrated Discovery (DAVID) (https://davidbioinformatics.nih.gov/summary.jsp). GO terms with a *p*‐value ≤ 0.05 were considered significant. To ensure specificity, overlapping GO terms between upregulated and downregulated DEG sets were excluded. For visualisation, enriched GO terms were plotted using ggplot2 (v3.4.4) in R, where the *X*‐axis represents –log_10_(*p*‐value) and the *Y*‐axis displays GO term descriptions. Dot colour intensity corresponds to fold enrichment.

### 4.10 Accession Numbers

The gene in this study has been submitted to the GenBank databases under accession numbers: *PaTAC1‐1* (ON565688), *PgTAC1‐1* (ON565689), *PaTAC1‐2* (ON565690), *PgTAC1‐2* (ON565691), PnTAC1 ‐ 1. ΔPnTAC1‐1 (ON565692) and *PnTAC1‐2* (ON565693).

## Author Contributions

Conceptualization: N.‐Y.C., M.‐H.K., E.‐K.B. and J.‐H.K. Performed the experiments and analysed the data: N.‐Y.C., M.‐H.K., H.‐A.J., S.‐W.P., K.‐Y.P., H.L., E.‐K.B. and J.‐H.K. Data curation: N.‐Y.C., M.‐H.K., H.‐A.J., S.‐W.P., K.‐Y.P. and H.L. Writing – original draft preparation: N.‐Y.C., M.‐H.K., E.‐K.B. and J.‐H.K. Writing – review and editing: N.‐Y.C., M.‐H.K., H.‐A.J., S.‐W.P., K.‐Y.P., H.L., E.‐K.B. and J.‐H.K. Funding acquisition: J.‐H.K. All authors have read and agreed to the published version of the manuscript.

## Conflicts of Interest

The authors declare no conflicts of interest.

## Supporting information


**Figures S1–S10:** pbi70415‐sup‐0001‐FigureS1‐S10.docx.

**Table S1:** pbi70415‐sup‐0002‐TableS1.xlsx.

**Table S2:** pbi70415‐sup‐0003‐TableS2.xlsx.

**Table S3:** pbi70415‐sup‐0004‐TableS3.xlsx.

## Data Availability

The data that support the findings of this study are available on request from the corresponding author. The data are not publicly available due to privacy or ethical restrictions.
