# Supplementary material for: Elucidating the Genetic Basis of Columnar Upright Architecture in Populus Through CRISPR Disruption of TILLER ANGLE CONTROL1
Source: Plant Biotechnol J. 2025 Oct 22;24(3):1377–97. doi: 10.1111/pbi.70415 (PMC12946507; doi:10.1111/pbi.70415)
Supplement: Supplementary file 1 — Figures S1–S10: pbi70415‐sup‐0001‐FigureS1‐S10.docx. [file PBI-24-1377-s002.docx]

**Title**:

**Elucidating the genetic basis of columnar upright architecture in *Populus* through CRISPR disruption of *TILLER ANGLE CONTROL1***

**Running title:**

***TAC1-CRISPR* produces upright architecture in poplar**

**Author names and affiliations:**

Na-Young Choi^1§^, Min-Ha Kim^1§^, Hyun-A Jang^1,2^, Seung-Won Pyo^1^, Kong-Young Park^3^, Hyoshin Lee^2^, Eun-Kyung Bae^2*^, and Jae-Heung Ko^1*^

^§^, These authors contributed equally.

**Figure S1. Comparative sequence and phylogenetic analysis of *TAC1* homologs in poplar and Arabidopsis.**

(a) Multiple sequence alignment of TAC1 protein sequences from *P. trichocarpa* (PtrTAC1-1: Potri.014G102600, PtrTAC1-2: Potri.002G175300), *P. alba* (PaTAC1-1: ON565688, PaTAC1-2: ON565690), *P. glandulosa* (PgTAC1-1: ON565689, PgTAC1-2: ON565691), and *P. nigra* var. *italica* (PnTAC1-1: ON565692, PnTAC1-2: ON565693), along with *A. thaliana* TAC1 (AtTAC1: AT2G46640.3). The truncated ΔPnTAC1-1 protein exhibits a premature stop codon resulting in a 61-amino-acid product (indicated by a red asterisk). The conserved IGT motif, characteristic of the IGT gene family, is highlighted in a blue box. (b) Phylogenetic tree constructed from the full-length amino acid sequences using the Neighbor-Joining method in MEGAX with 1,000 bootstrap replicates and the JTT substitution model. The tree demonstrates clustering of TAC1 homologs into two major clades, with ΔPnTAC1-1 grouping distinctly due to its truncated sequence.

**Figure S2. Confirmation of the nonsense mutation in *PnTAC1-1* from Lombardy poplar clones.**

Four-color Sanger sequencing chromatograms showing partial nucleotide sequences of *TAC1-1* orthologs from BH poplar (*PaTAC1-1* and *PgTAC1-1*) and two trees of Lombardy poplar clones (*PnTAC1-1*). The red box marks a point mutation in *PnTAC1-1*, where a thymine-to-adenine substitution converts the leucine codon (TTA) to a premature stop codon (TAA). Blue asterisks indicate the location of the mutated base in both sampled trees of male *PnTAC1-1* alleles.

**Figure S3. Design of single guide RNAs (sgRNAs) targeting *TAC1* genes in hybrid poplar (*P. alba* × *P. glandulosa*, clone BH).**

(a) CRISPR sgRNA target sites were designed using Cas-Designer from the CRISPR RGEN Tools platform (http://www.rgenome.net/cas-designer). Two sgRNAs (sg1 and sg2) were selected to target the third exon of all four TAC1 homologs—*PaTAC1-1*, *PaTAC1-2*, *PgTAC1-1*, and *PgTAC1-2*. The sgRNAs are separated by 297 bp in *PaTAC1-1*/*PgTAC1-1* and 327 bp in *PaTAC1-2*/*PgTAC1-2*. Each target site was evaluated for GC content, direction, out-of-frame score, and number of potential off-target mismatches. Selected sgRNAs had high out-of-frame scores (≥72) and minimal predicted mismatches. (b) Summary of sgRNA sequences used in this study. Both sg1 and sg2 target all four *TAC1* alleles. Protospacer adjacent motif (PAM) sequences are underlined.

**Figure S4. Generation and transgene confirmation of *CRISPR-TAC1* hybrid poplars.**

(a) Regenerated hybrid poplar shoots following *Agrobacterium*-mediated transformation with the CRISPR/Cas9–PtrTAC1 construct. Surviving shoots are shown in test tubes, with confirmed transgenic lines (#8, #24, #25, #26, and #27) highlighted in red. Wild-type BH1 and BH2 served as negative controls. (b) PCR confirmation of transgene integration. Primer sets targeting the hygromycin phosphotransferase gene (Hyg; 514 bp) and the CaMV 35S promoter (35S; 456 bp) verified transgene presence, while BH1 and BH2 were negative controls and plasmid DNA (PL) was a positive control. Poplar *ACTIN2* gene (PtrACTIN2; 753 bp) was amplified as an internal control for genomic DNA quality. This panel combines two separate gel images, with yellow dividing lines marking the boundaries between distinct gels.

**Figure S5. Mutated gene structure of the selected *TAC1-CRISPR* hybrid poplar lines.**

Schematic diagrams show the exon-intron structures of the four TAC1 homologs (*PaTAC1-1*, *PaTAC1-2*, *PgTAC1-1*, and *PgTAC1-2*) from the hybrid poplar BH line and their corresponding CRISPR-edited alleles (Type-1 and Type-2). Single guide RNAs (sg1 and sg2) were designed to target the third exon (white boxes indicate sgRNA target sites). CRISPR/Cas9 editing resulted in frameshift mutations that introduced premature stop codons at the indicated nucleotide positions (numbered from ATG). Scale bar = 0.5 kb.

**Figure S6. Genotyping of *TAC1-CRISPR* replicon poplars.**

(a) Genomic DNA-PCR analysis results of *TAC1-CRISPR* replicon transgenic poplars (6-week after rooting). ‘T-DNA’ indicates to confirm the T-DNA insertion in the poplar genome using primer sets targeting the hygromycin phosphotransferase gene (Hyg; 514 bp). To check *TAC1* gene mutation, sg1 and sg2 target sequences were used as primer pair. *PtrACTIN2* was used as a loading control. This panel combines two separate gel images, with yellow dividing lines marking the boundaries between distinct gels. (b) Indel mutations of the *TAC1-CRISPR* replicon poplar lines. Identified indels in target sequences of each line are highlighted in red-colored letters or line (insertion) or blue lines (deletion), and the corresponding indel numbers are shown on the right (‘-’ blue numbers, deletion; ‘+’ red numbers, insertion). Note the red line with yellow shading emphasizes a large insertion. (c) Summary of mutations in *TAC1-CRISPR* hybrid poplars. Expected normal TAC1 proteins (PaTAC1-1/2 and PgTAC1-1/2) in the control (BH) are shown as black bars with corresponding sizes. TAC1 proteins mutated by CRISPR editing are shown as gray bars with corresponding sizes. Asterisks indicate non-sense mutations and the number of amino acids is shown in the right.

**Figure S7. *TAC1-CRISPR* replicon poplars showed upright growth architecture.**

(a) *TAC1-CRISPR* replicon poplar plants grown in LMO field for 6 weeks showing narrow branch angles. Photograph was taken on June 21, 2022. (b ~ d) Quantification of phenotypic characteristics of (a) plants. Stem height (b), stem diameter (c), and branch angle (d). N = 30, Error bars = S.D.

**Figure S8. Validation of transcriptome data by qRT-PCR analysis.**

(a, b) Quantitative real-time RT-PCR analysis of selected genes in axillary meristem (a) and shoot apical meristem (b) tissues from BH and *TAC1-CRISPR* lines (#24 and #26).

Blue lines represent relative gene expression levels obtained by qRT-PCR, normalized to *PtrACTIN2*, while black bars indicate corresponding transcript abundance from RNA-seq (TPM values). The qRT-PCR results generally align with RNA-seq data, supporting the reliability of the transcriptomic analysis. Genes analyzed include the TAC1 homologs — *PtrTAC1-1* (Potri.014G102600) and *PtrTAC1-2* (Potri.002G175300) — as well as meristem and cell wall-associated genes: *PtrANT* (Potri.005G148400), *PtrPME* (Potri.001G119300), *PtrCesA4* (Potri.002G257900), *PtrCesA7* (Potri.018G103900), *PtrCesA8* (Potri.011G069600), *PtrPAL1* (Potri.006G126800), *PtrCCR2* (Potri.003G181400), *PtrC4H1* (Potri.013G157900), *PtrCAD1* (Potri.009G095800), *PtrCOMT2* (Potri.012G006400), *PtrF5H1* (Potri.007G016400), and *PtrCCoAOMT1* (Potri.001G304800).

**Figure S9. GO enrichment analysis of downregulated DEGs in *TAC1-CRISPR* lines.**

GO enrichment analysis of downregulated DEGs in AM tissues of *TAC1-CRISPR* lines, categorized by biological processes. Only non-overlapping GO terms (P ≤ 0.05) are shown. Dot size corresponds to the number of genes, while color intensity reflects fold enrichment, calculated as the ratio of gene frequency in the query set to that in the background.

**Figure S10. Expression of *TAC1* genes in shoot apical meristem (SAM) and axillary meristem (AM) tissues of *TAC1-CRISPR* and BH poplars.**

RNA-seq-based transcript levels (TPM) of *PtrTAC1-1* (Potri.014G102600) and *PtrTAC1-2* (Potri.002G175300) are shown for SAM and AM tissues of BH (wild type) and *TAC1-CRISPR* lines (#24 and #26).


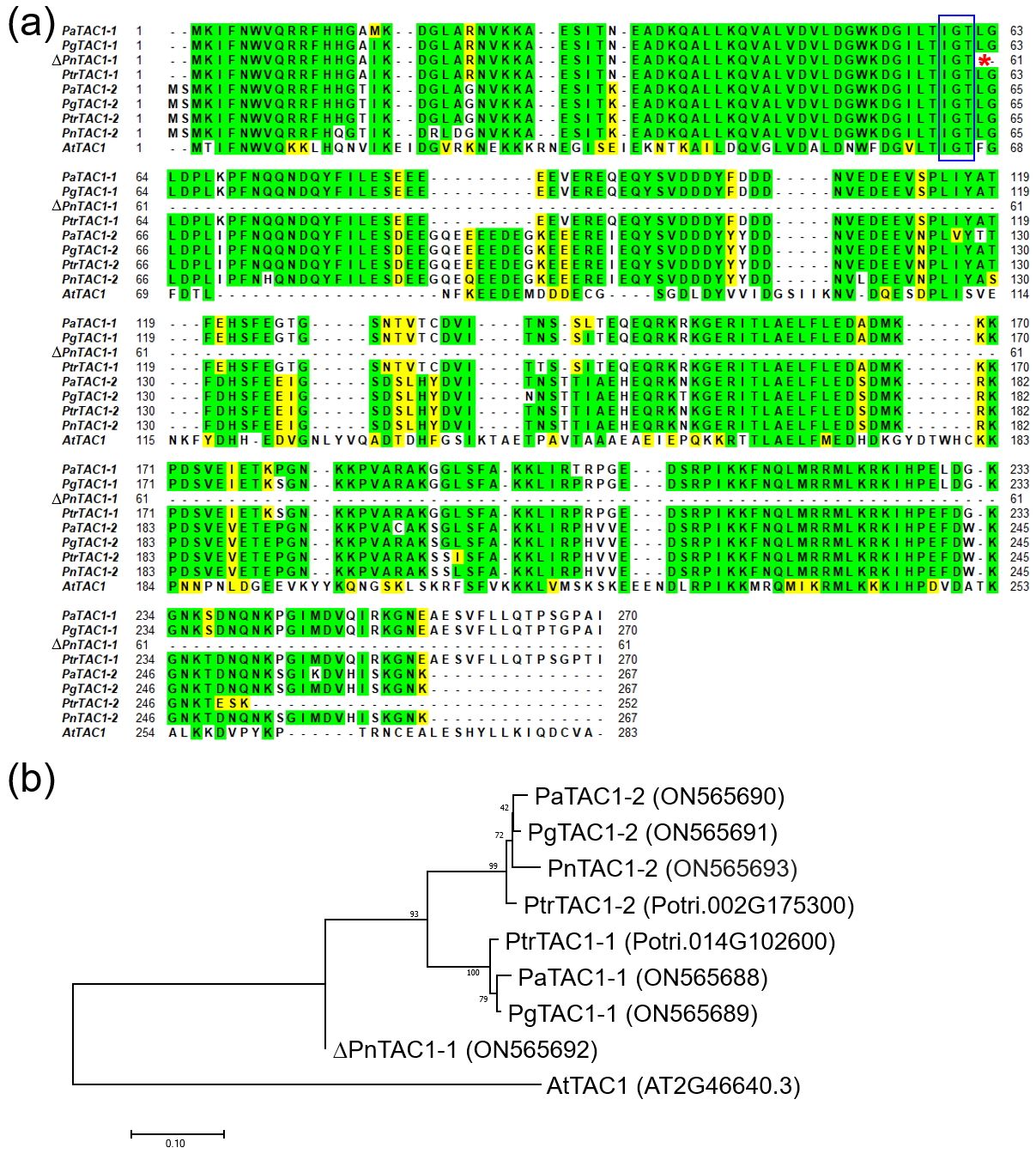


**Figure S1. Comparative sequence and phylogenetic analysis of TAC1 homologs in poplar and Arabidopsis.**


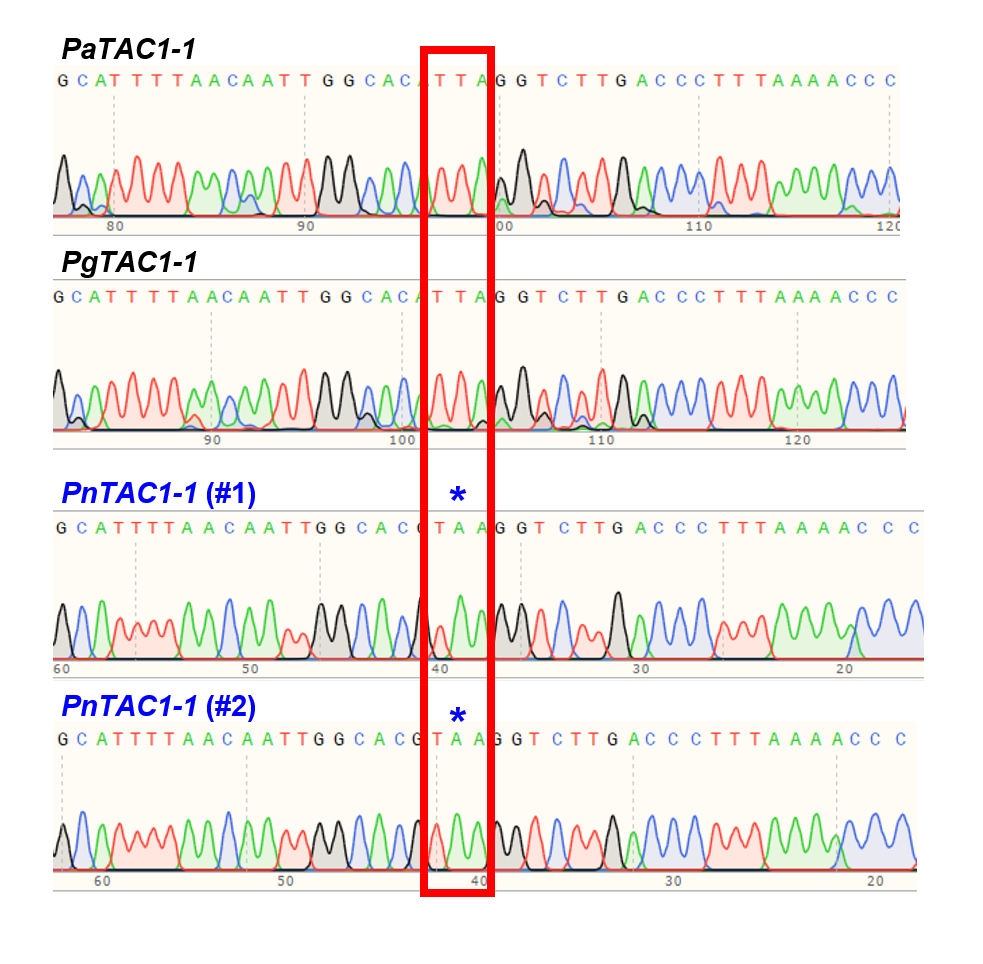


**Figure S2. Confirmation of the nonsense mutation in PnTAC1-1 from Lombardy poplar clones.**


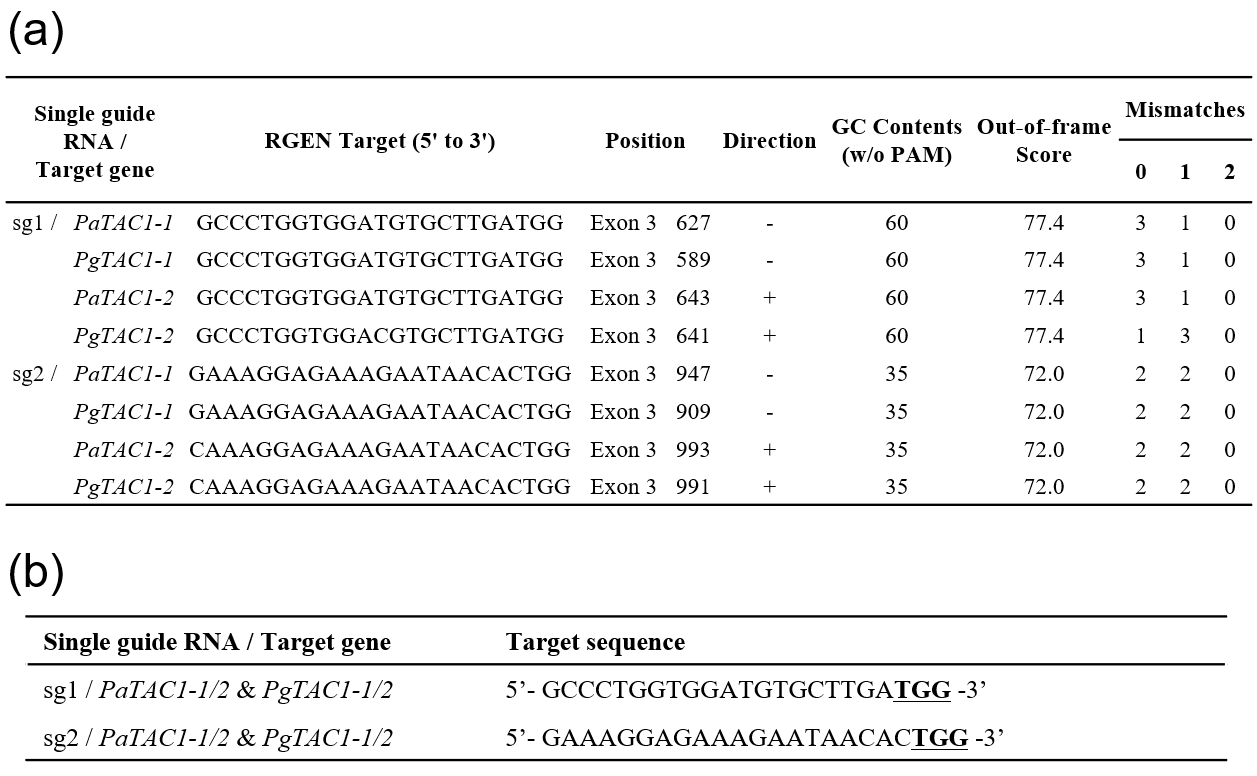


**Figure S3. Design of single guide RNAs (sgRNAs) targeting *TAC1* genes in hybrid poplar (*P. alba* × *P. glandulosa*, clone BH).**


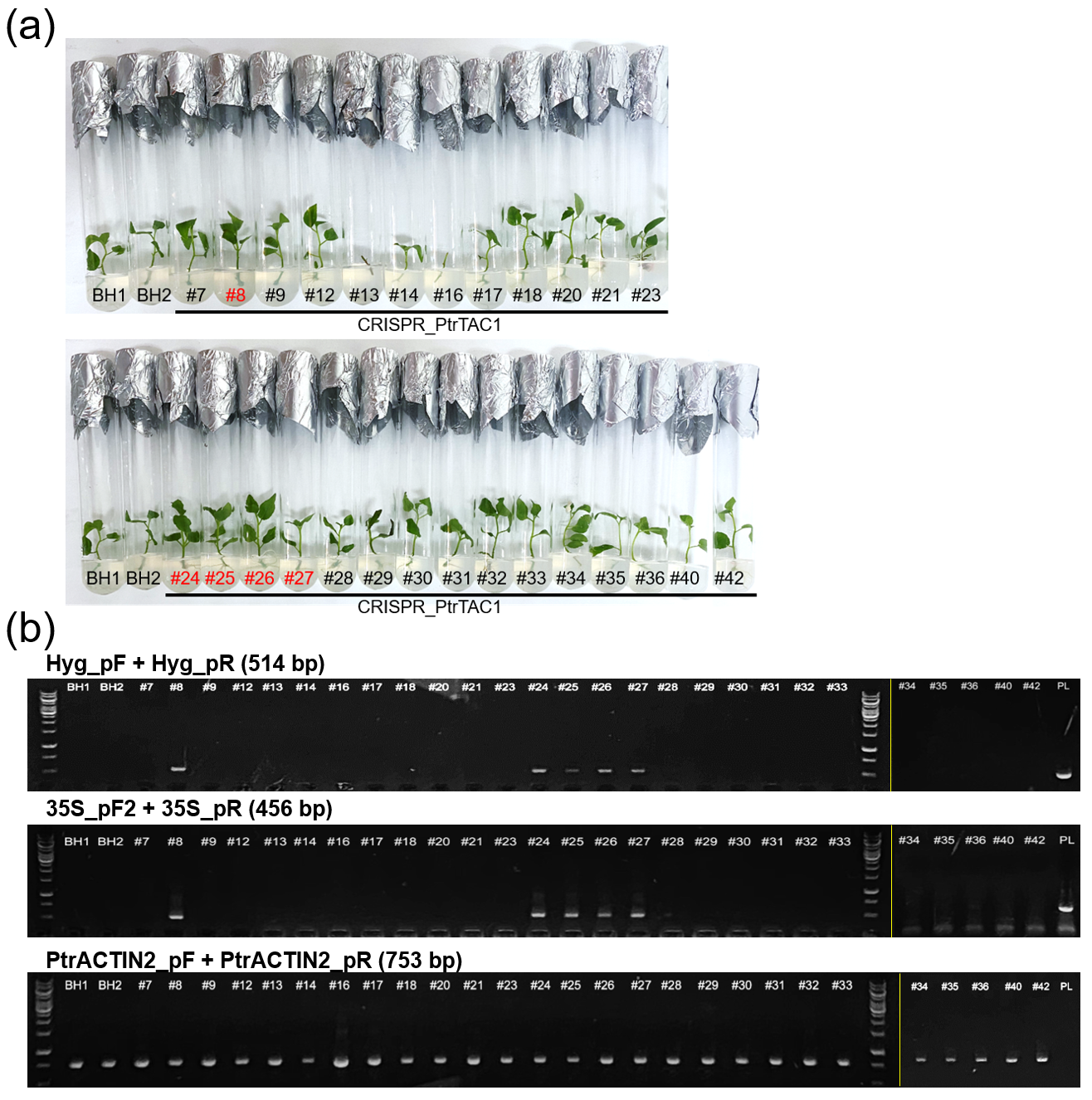


**Figure S4. Generation and transgene confirmation of *CRISPR-TAC1* hybrid poplars.**


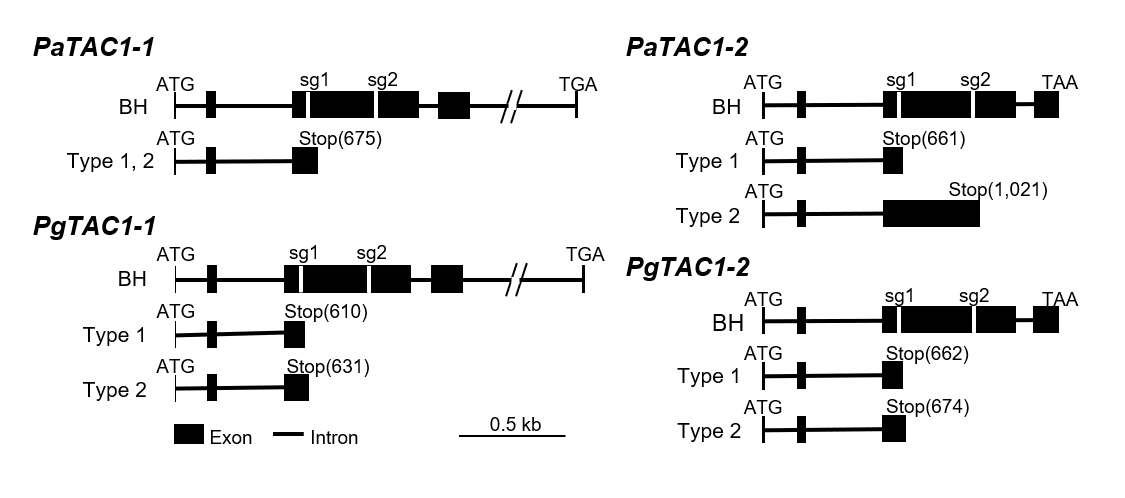


**Figure S5. Mutated gene structure of the selected *TAC1-CRISPR* hybrid poplar lines.**


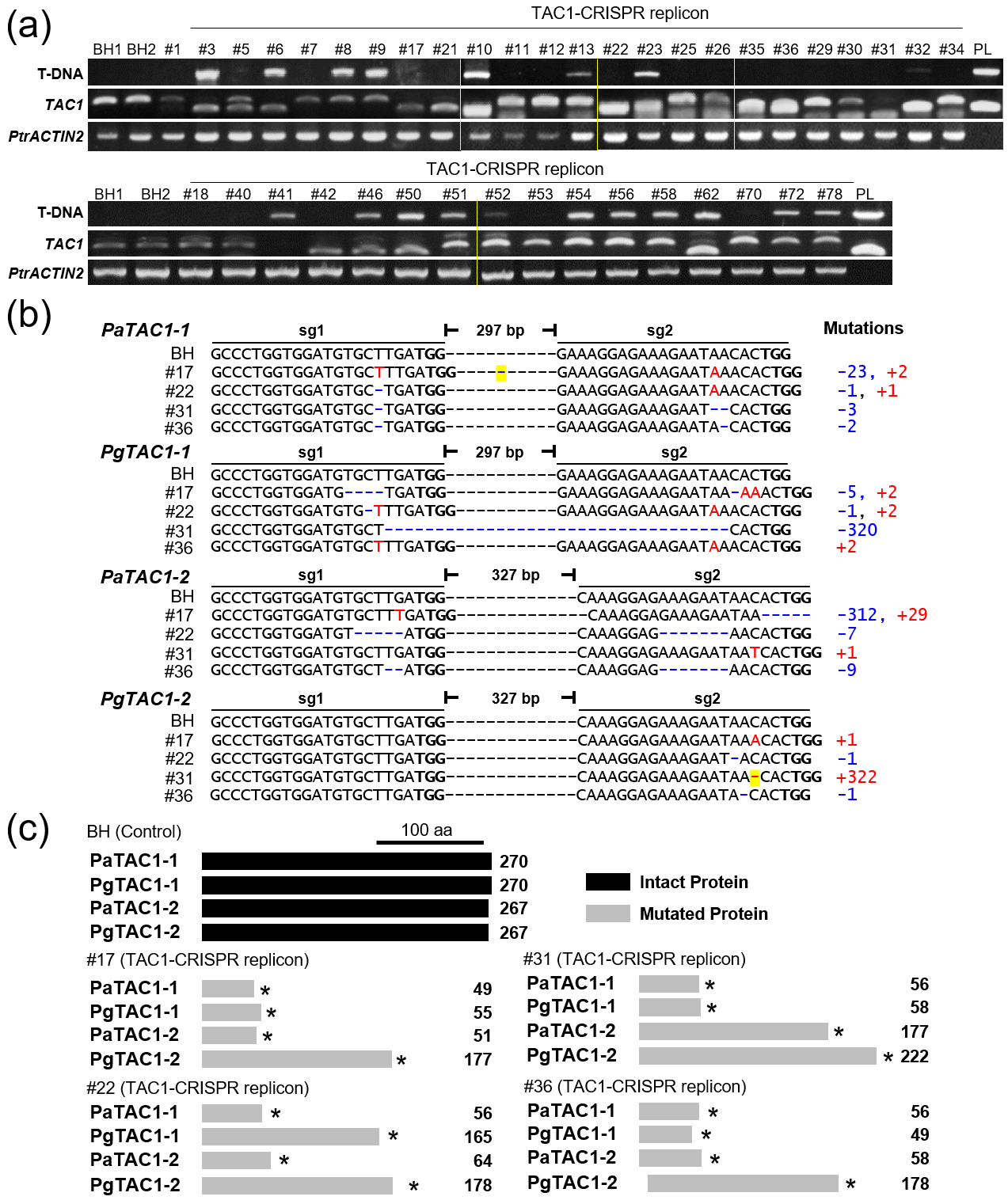


**Figure S6. Genotyping of TAC1-CRISPR replicon poplars.**


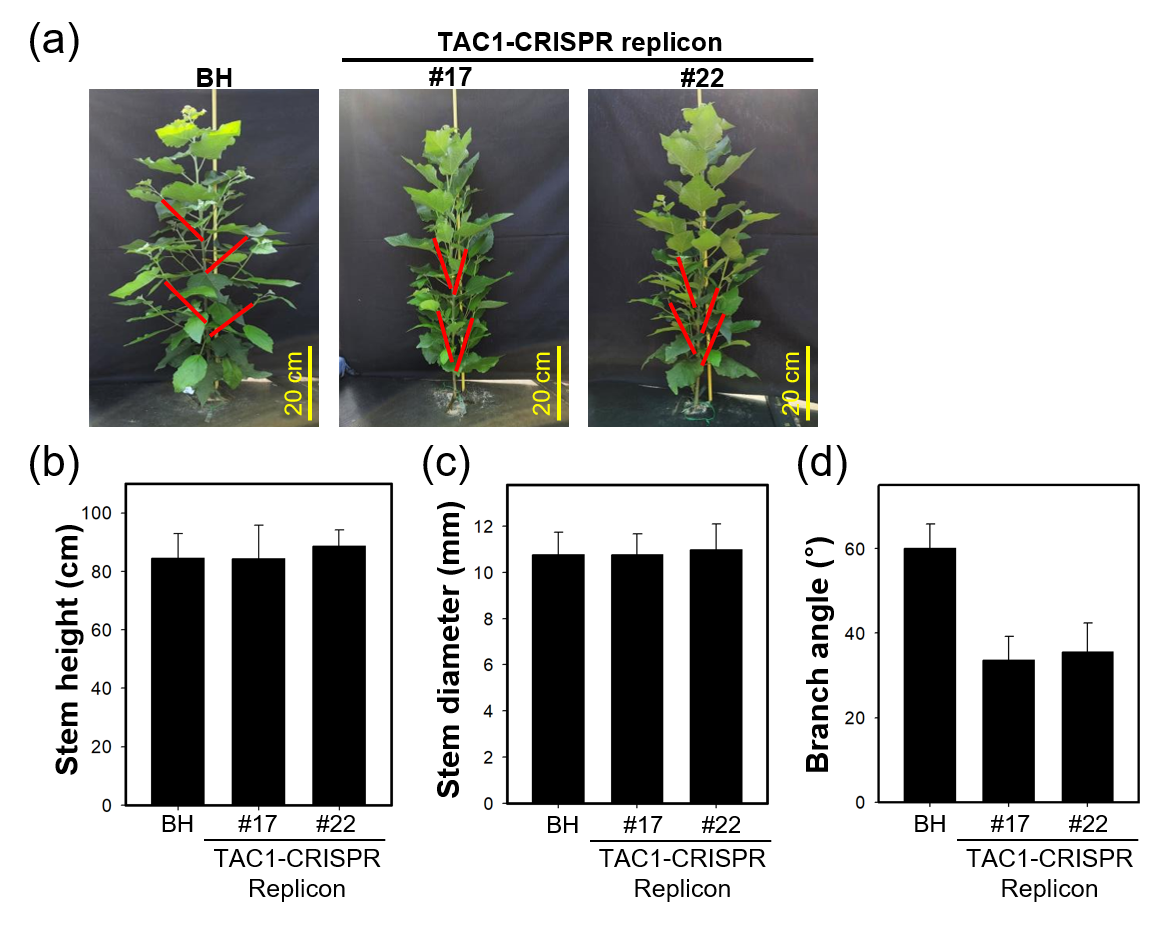


**Figure S7. *TAC1-CRISPR* replicon poplars showed upright growth architecture.**


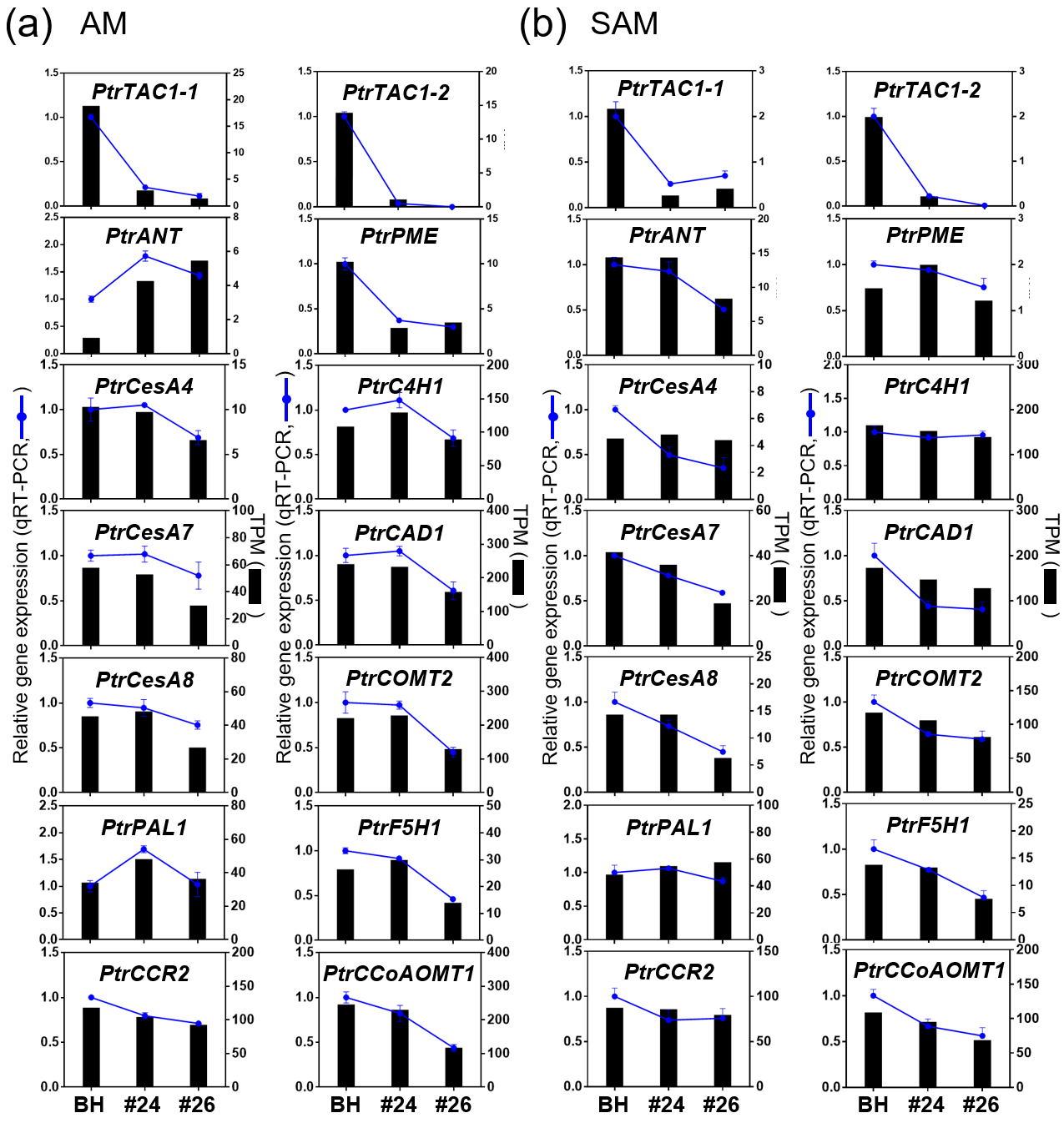


**Figure S8. Validation of transcriptome data by qRT-PCR analysis.**


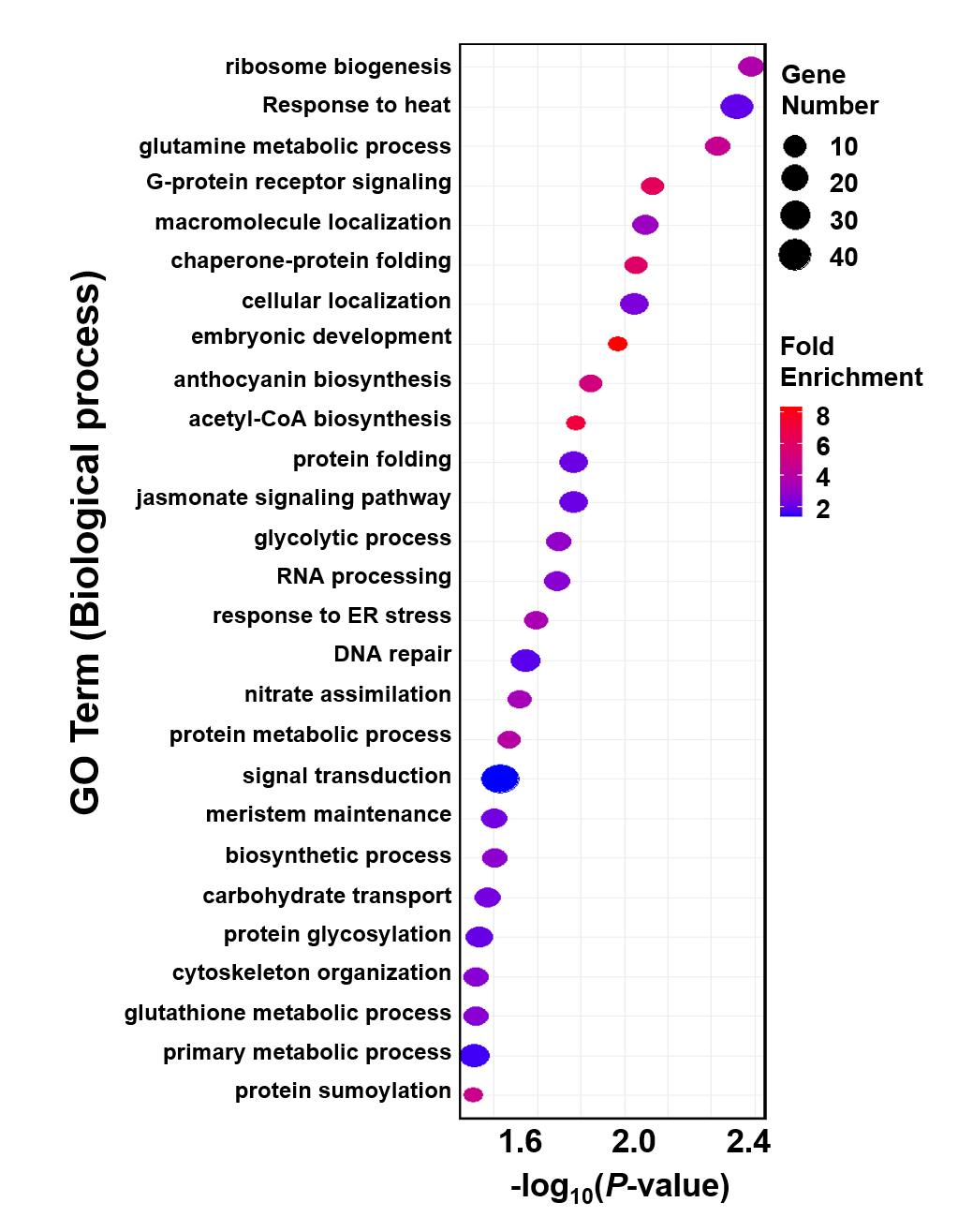


**Figure S9. GO enrichment analysis of downregulated DEGs in *TAC1-CRISPR* lines.**


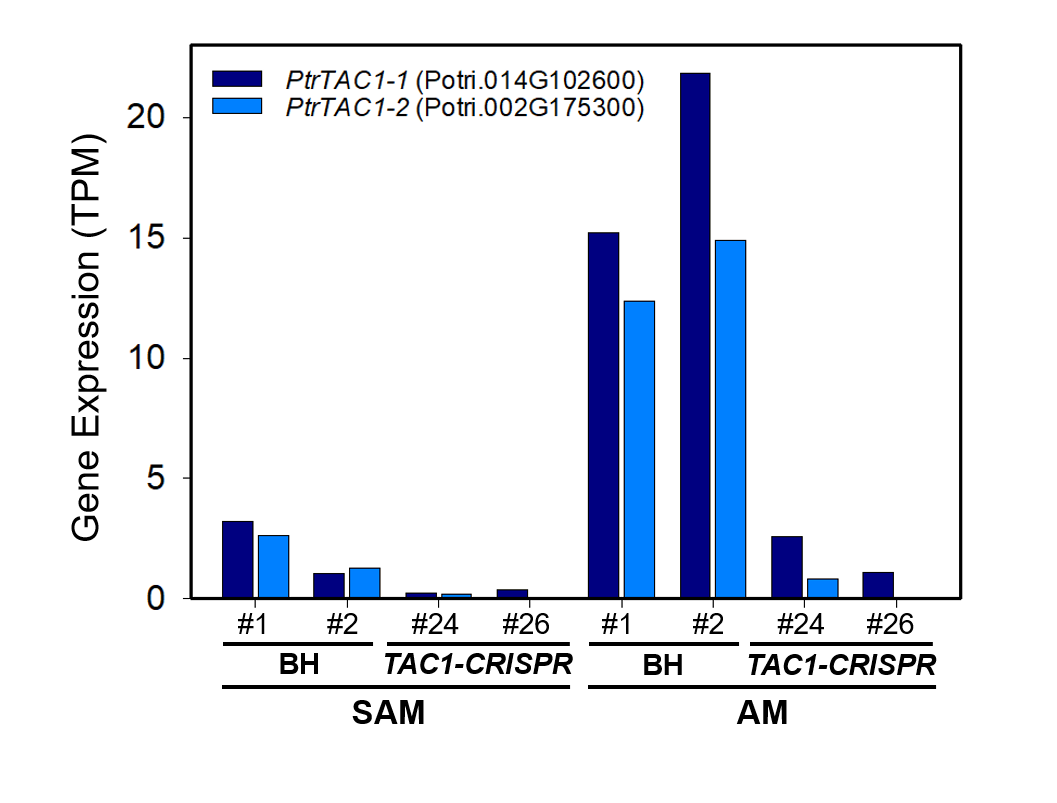


**Figure S10. Expression of *TAC1* genes in shoot apical meristem (SAM) and axillary meristem (AM) tissues of *TAC1-CRISPR* and BH poplars.**
